# Enhancing the Pharmaceutical Profile of Alpha Lipoic Acid: Cyclodextrin Inclusion Complexation for Improved Stability and Bioavailability

**DOI:** 10.3390/pharmaceutics18070780

**Published:** 2026-06-25

**Authors:** Karolina Miljak, Kristina Radić, Emerik Galić, Vedrana Špada, Lucija Vrban Đerek, Robert Vianello, Dubravka Vitali Čepo, Mario Jug

**Affiliations:** 1 University of Zagreb Faculty of Pharmacy and Biochemistry, Department of Nutrition and Dietetics, A. Kovačića 1, 10000 Zagreb, Croatia; kmiljak@student.pharma.hr (K.M.); kradic@pharma.hr (K.R.); egalic@pharma.hr (E.G.); dvitali@pharma.hr (D.V.Č.); 2 Istrian University of Applied Sciences, METRIS Research Centre, Preradovićeva 9D, 52100 Pula, Croatia; vspada@iv.hr; 3 Ruđer Bošković Institute, Laboratory for the Computational Design and Synthesis of Functional Materials, Bijenička 54, 10000 Zagreb, Croatia; lucija.vrban@irb.hr (L.V.Đ.); robert.vianello@irb.hr (R.V.); 4 University of Zagreb Faculty of Pharmacy and Biochemistry, Department of Pharmaceutical Technology, A. Kovačića 1, 10000 Zagreb, Croatia

**Keywords:** alpha lipoic acid, solubility, cyclodextrin, inclusion complexes, chemical stability, in vitro permeability

## Abstract

**Background/Objectives**: α-lipoic acid (ALA) shows therapeutic potential but faces poor aqueous solubility (BCS Class II), gastric instability, and low oral bioavailability (~30%). This work investigated the formulation of cyclodextrin (CD) inclusion complexes of ALA to overcome the aforementioned limitations and improve nutraceutical applications. **Methods**: Phase solubility studies in simulated gastric and intestinal fluids screened for optimal CD, followed by molecular dynamics simulations and MM-PBSA binding free energy calculations. Inclusion complexes of choice were prepared by grinding, spray-drying, and lyophilization, followed by solid-state characterization (DSC/XRPD/FTIR). Further analysis was performed using pH-shift dissolution (USP II), permeability (PermeaPad^®^, Caco-2), and (photo)stability according to ICH. **Results**: Hydroxypropyl-β-cyclodextrin (HPβCD) emerged as the optimal host due to favorable complexation, as confirmed by phase solubility studies and supported by molecular modeling, which revealed a favorable balance between inclusion complex stability and pH-triggered drug release. Formulations based on spray-dried and lyophilized HPβCD–ALA complexes (HPβALA-sd and HPβALA-lyo), in which ALA was fully amorphized, achieved near-complete dissolution within five minutes under biorelevant pH-shift conditions. This performance markedly exceeded that of free ALA (approximately 66% dissolution at pH 7.4) while maintaining moderate permeability (Papp 8–9 × 10^−6^ cm/s). Storage stability was enhanced markedly (88–90% ALA retention after 6 months at 40 °C/75% RH vs. 36% for free ALA) while UV stability was not improved through CD-complexation, probably due to interaction of UV-VIS light with the exposed portion of ALA. **Conclusions**: Even though the permeability of ALA–CD inclusion complexes remained medium (Papp ~ 8–9 × 10^−6^ cm/s) and unaffected by complexation, a significantly improved dissolution profile indicates better expected bioavailability compared to pure ALA.

## 1. Introduction

α-lipoic acid (ALA) is an organosulfur compound, also known as 1,2-dithiolane-3-pentanoic acid or thioctic acid, essential for various enzymatic functions, primarily in mitochondria. Even though it can be synthesized within the human body, the majority of ALA is obtained from the diet, particularly meat and vegetables. As an enzymatic cofactor (e.g., pyruvate dehydrogenase complex and α-ketoglutarate dehydrogenase complex involved in energy production in mitochondria) and strong antioxidant, it is involved in the regulation of glucose and lipid metabolism and regulation of gene transcription [1].

In recent decades, ALA (including its reduced form, dehydro-α-lipoic acid has attracted substantial interest due to its therapeutic benefits in managing various conditions [2]. In numerous clinical studies, ALA has exhibited promising cardiovascular [3,4], cognitive [5,6,7], anti-aging [8], detoxifying [9], antioxidant and anti-inflammatory [10,11,12,13], anti-cancer [14,15], and neuroprotective [13,16] effects.

ALA faces significant pharmacokinetic and stability challenges that limit its clinical utility, particularly when administered orally. Clinical studies consistently report low oral bioavailability, around 30%, a short plasma half-life (25–33 min), and rapid clearance due to gastric instability, poor solubility, and extensive first-pass hepatic metabolism [1]. Oral ALA exhibits rapid but incomplete absorption, with peak plasma concentration (C_max_) reached in 30–60 min post-dose, yet absolute bioavailability remains low at 29.1% for 200 mg doses in healthy volunteers. A randomized crossover trial in 12 subjects showed area under the curve values of 46.82 μg·min/mL for 200 mg oral versus 157.97 μg·min/mL intravenous, confirming substantial presystemic losses. High inter- and intra-individual variability in C_max_, noted in recent Phase 1 trials, stems from ALA’s low solubility (class II by biopharmaceutics classification system (BCS)), complicating dose predictability in patients [1,17,18,19].

Due to its asymmetric carbon atom, ALA exists as two optical isomers. The naturally occurring R-enantiomer of ALA is the biologically active form produced endogenously, while the S-enantiomer lacks these endogenous roles and shows reduced potency. The R-enantiomer displays superior pharmacokinetics over racemic mixtures, achieving higher C_max_ (e.g., 8 μg/mL at 300 mg liquid dose) and area under the curve (178 min·μg/mL) in fasting healthy volunteers, though elimination half-life remains brief (~6–30 min), restricting sustained therapeutic levels [19].

However, most commercial ALA supplements contain racemic mixtures (50:50 R:S) [1]. This preference stems from the simplicity of producing racemates through straightforward chemical synthesis, whereas isolating pure R-ALA requires complex, costly separation techniques, such as chemical or enzymatic resolution of racemic intermediates. Moreover, limited evidence indicates that the S-enantiomer stabilizes the R-enantiomer in formulations by inhibiting polymerization [20]. The proposed stabilization of R-lipoic acid by the S-enantiomer has not been fully elucidated, but it is probably related to altered solid-state organization, intermolecular interactions, or formation of dihydrolipoic acid and subsequent disulfide bridge formation [21,22].

ALA’s dithiolane ring is prone to polymerization and oxidative degradation, especially under thermal (>48 °C), acidic, or oxidative conditions, yielding sulfurous byproducts and reduced bioactivity. Forced degradation studies reveal instability in acidic/basic/peroxide environments, with liquid formulations offering modest protection via stabilizers, though solid tablets exacerbate dissolution delays [17].

Poor organoleptic properties (e.g., unpleasant odor and taste), chemical instability, and low aqueous solubility of bioactive compounds of ALA pose substantial barriers to their effective oral delivery and therapeutic efficacy. These challenges, rooted in ALA’s susceptibility to oxidative polymerization, pH-dependent degradation, and BCS Class II profile, can be effectively mitigated through advanced drug formulation strategies, formulation of solid lipid nanoparticles (SLNs), emulsification, liposomes, and formulation of inclusion complexes—particularly with cyclodextrins (CDs) [23].

Even though SLNs, composed of physiological lipids, address ALA’s lipophilicity by forming nanoscale matrices (50–200 nm) that boost lymphatic uptake and increase bioavailability [24,25], formulation of CD-based inclusion complexes with ALA might offer distinct advantages over lipid-based systems, particularly in terms of technological simplicity, scalability, cost-effectiveness, and ecological footprint [26,27]. Furthermore, CD–ALA complexes might provide superior physical stability without the risk of lipid peroxidation or Ostwald ripening observed in emulsions and liposomes, achieving solubility enhancement through non-covalent encapsulation of ALA’s dithiolane moiety, independent of complex surfactants that may cause gastrointestinal irritation in emulsions. Unlike SLNs, which can suffer from drug expulsion during storage due to lipid polymorphism [28,29], CD complexes maintain amorphous ALA dispersion with consistent 1:1 stoichiometry, facilitating faster dissolution and predictable pharmacokinetics [30,31]. Several investigations have also incorporated a ternary component—such as polymers, hyaluronic acid, or amino acids—to further improve solubility, stability, or targeted or sustained delivery [26,28,29,30,31,32].

CDs are cyclic oligosaccharides featuring a hydrophilic exterior and hydrophobic cavity that accommodates poorly soluble drugs, thereby enhancing their pharmacokinetic profiles and/or therapeutic efficacy. They are widely regarded as safe excipients for oral drug delivery due to their limited gastrointestinal absorption and low systemic exposure. Following oral administration, γCD is digested by α-amylases, whereas αCD and βCD and their hydrophilic derivatives (including HPβCD) are primarily metabolized by colonic microbiota. Their high molecular weight, hydrophilicity, and extensive hydrogen-bonding capacity restrict passive membrane permeation, resulting in oral bioavailability typically below 3% [33,34]. Consequently, CDs are considered practically non-toxic when administered orally, with diarrhea as the main adverse effect at high doses due to osmotic activity [33]. While certain safety concerns, such as nephrotoxicity and membrane interactions, have been reported for specific CDs and routes of administration (notably parenteral), these effects are not relevant for oral delivery [34]. Selected CDs, including HPβCD, are well-established excipients with regulatory approval (GRAS status, pharmacopoeial monographs, FDA Inactive Ingredient Database), although challenges related to substitution heterogeneity and formulation design remain important considerations [33,34].

CD inclusion complexation can be further elucidated using molecular modeling approaches, which provide insight into host–guest interactions, binding modes, and the influence of environmental factors such as pH [35,36]. Such approaches are particularly relevant for ALA due to its amphiphilic structure and pH-dependent ionization, which may significantly influence its interaction with CDs.

As shown in Table 1, several authors have explored ALA inclusion complexes with various CDs, including native types and synthetic derivatives. Complexes were prepared using various technologies (kneading, freeze-drying, evaporation, coprecipitation, and electrospinning), typically to improve the thermal stability or solubility/bioavailability of ALA.

While existing research on ALA–CD inclusion complexes, summarized in Table 1, has laid foundational groundwork by exploring various CDs and preparation methods, it underscores the need to upgrade these investigations due to pervasive gaps in comprehensive stability and practical utility data. Table 1 reveals predominantly partial characterizations, with many studies limited to basic techniques focusing narrowly on either solubility enhancements or, in some cases, thermal stability, while omitting critical metrics like long-term storage stability or release kinetics under physiological conditions. Moreover, key preparation methods remain underexplored, including spray-drying, hot-melt extrusion, supercritical fluid processing, or microfluidics, which could yield more scalable, amorphous dispersions with superior dissolution profiles compared to the rudimentary approaches tabulated. Additionally, no studies have investigated ternary component systems, which are well-established in the literature for synergistically boosting complexation efficiency, inhibiting crystallization, and enhancing muco-adhesion or permeation [37].

This research builds upon existing findings and will focus on the investigation of the feasibility of preparing ALA–CD inclusion complexes via spray-drying and solid-state ALA–CD inclusion complex preparation using mechanical micro-milling. Spray-drying offers superior potential for industrial scalability compared to alternative approaches but has not been systematically explored to date. Solid-state inclusion complex preparation offers an environmentally friendly alternative to other methods that remain unexplored to date. ALA–CD inclusion complexes will be thoroughly characterized, focusing on a broad range of technological and biopharmaceutical parameters, particularly gastrointestinal stability and permeability, key determinants of oral bioavailability that have not been previously investigated. Additionally, the development of multicomponent systems based on ALA–CD inclusion complexes to further enhance their technological and biopharmaceutical properties will be investigated. The research will emphasize CDs with *generally recognized as safe* status to ensure applicability in novel food and nutraceutical formulations.

**Table 1:** Investigation of properties of ALA–CD complexes prepared by different technologies.

Cyclodextrin	Technology of Preparation	Characterization	Target Functionality Aspect	Effect	Ref.
βCD	-	NMR	molecular modeling	↗ solubility	[38]
βCD; HPβCD mG1-βCD Me-βCD DMe-βCD	-	NMR	solubility	↗ solubility	[39]
SBE-βCD	1:1; kneading	NMR, FTIR, DSC, XRD	solubility	↗ solubility (20× when compared to pure ALA)	
	1:1; freeze-drying		thermal stability	↗ thermal stability	
ɑCD βCD γCD	20 g:180 g; freeze-drying	DSC	solubility	no effect	[40]
mG2-αCD mG2-βCD mG2-yCD				↗ solubility	
ɑCD βCD γCD	20 g:180 g; freeze-drying	DSC	thermal stability	↗ thermal stability	[40]
mG2-αCD mG2-yCD				no effect	
mG2-βCD				↗ thermal stability	
γCD	40 g:160 g; freeze-drying	-	bioavailability (rats)	no effect	[41]
βCD	1:1; evaporation	XRD, FTIR, NRM, DSC	molecular spectroscopy	no effect	[42]
	1:1; freeze-drying				
ɑCD βCD γCD	1:1; freeze-drying	DSC, XRD	thermal stability; stability under acidic conditions	↗ thermal stability; ↗ stability under acidic conditions	[20]
ɑCD βCD γCD	-	FTIR, Raman	spectroscopic mapping	no effect	[43]
ɑCD	2:3; co-precipitation	XRD, FTIR, Raman, NMR	structural analysis	no effect	[44]
ɑCD βCD	1:1	NMR	-	-	[45]
γCD	2:1				
ɑCD βCD	1:6.71 mg; 1:7.81 mg	-	bioavailability (rats)	no effect	[46]
γCD	1:8.77 mg			↗ bioavailability	
γCD	1:1	-	bioavailability (humans)	↗ bioavailability	[47]
HXm-ɑCD HEPm-βCD	1:1; co-precipitation	DSC, TGA	-	-	[48]
HPβCD HPγCD	1:1; 2:1; nanofibers	NMR, FTIR, DSC, XRD, TGA	solubility	↗ solubility	[49]

mG1-βCD—mono-6-O-glucopyranosyl-βCD; Me-βCD—methyl-βCD; DMe-βCD—2,6-di-O-methyl-βCD; SBE-βCD—sulfobutylether-βCD; mG2-α(βγ)CD—6-O-α-maltosyl-α(βγ)-CD; HXm-ɑCD—hexakis(2,3,6-tri-O-methyl)-ɑCD; HEPm-βCD—heptakis(2,3,6-tri-O-methyl)-βCD; HPβ(γ)CD—hydroxypropyl-β(γ)CD; DSC—differential scanning calorimetry; NMR—nuclear magnetic resonance; FTIR—Fourier-Transform Infrared Spectroscopy; XRD—X-ray diffraction; TGA—thermogravimetric analysis.

## 2. Materials and Methods

### 2.1. Chemicals

ALA (CAS: 1077-28-7)), αCD (CAS: 10016-20-3), βCD (CAS: 7585-39-9), γCD (CAS: 17465-86-0), 2-Hydroxypropyl-α-CD (HPαCD) (CAS: 99241-24-4, degree of substitution ≈ 4.8), 2-Hydroxypropyl-γ-CD (HPγCD) (CAS: 128446-34-4, degree of substitution 4.0–5.6) were obtained from Sigma Aldrich (St. Louis, MO, USA), and 2-Hydroxypropyl-β-CD (HPβCD) (CAS: 128446-35-5, degree of substitution ≈ 4.5) was obtained from Cyclolab (Budapest, Hungary).

Acetonitrile (CH_3_CN; ≥99.93%, HPLC grade), methanol (CH_3_OH; ≥99.9%, HPLC grade), Dulbecco’s Modified Eagle’s Medium (DMEM, Product Number: D6429), dimethyl sulfoxide (DMSO, CAS: 67-68-5), pepsin from porcine gastric mucosa (Pepsin, CAS: 9001-75-6), porcine bile extract (Bile salts, CAS: 8008-63-7), and pancreatin (8 USP, CAS Number: 8049-47-6), were obtained from Sigma-Aldrich. Potassium dihydrogen phosphate (KH_2_PO_4_; CAS 7778-77-0) was obtained from Gram-mol d.o.o. (Zagreb, Croatia) and 3-(4,5-Dimethylthiazol-2-yl)-2,5-diphenyltetrazolium bromide (MTT) reagent was sourced from Carbosynth Limited (Compton, UK). Ultrapure water was obtained using a Milli-Q Advantage A10 purification system (Merck; Darmstadt, Germany). All other chemicals were of p.a. grade and used as received.

Additionally, PES syringe filters (25 mm diameter, 0.22 µm pore size) from FilterBio, Labtex Ltd. (Jinan, China) were used to prepare samples for chromatographic analysis, and cellulose acetate filters (0.45 µm pore size) from Whatman (Merck) were used to filter the mobile phase.

### 2.2. Phase Solubility Study

Phase solubility studies were performed by adding increasing amounts of CD (αCD, βCD, γCD, HPαCD, HPβCD, HPγCD; 6–30 mM, in a final volume of 10 mL) to 20 mL glass vials. An excess of ALA (≈200 mg) was added to each vial, followed by 8 mL of 1.25× simulated gastric fluid (SGF) or 1.25× simulated intestinal fluid (SIF) prepared according to the INFOGEST 2.0 protocol [50], and water was added to reach 10 mL. Suspensions were sealed, protected from light, and shaken at 150 rpm and 37 °C. All experiments were performed in triplicate.

After 24 and 48 h, aliquots were collected, filtered, diluted 1:1 with methanol, and analyzed by high-performance liquid chromatography with diode array detection (HPLC-DAD) at 215 nm [51]. Phase-solubility diagrams were constructed by plotting ALA solubility versus CD concentration. Apparent stability constants (K_s_) were calculated using Equation (1):

(1) $$K_{s}=\frac{\mathrm{slope}}{c_{\mathrm{ALA}}(1-\mathrm{slope})}$$

where $c_{\mathrm{ALA}}$ is the saturation solubility of ALA in the absence of CD after 24 and 48 h. Phase solubility studies were also performed following the same experimental protocol by incorporating a suitable ternary component, selected based on an isothermal compatibility test (Section 2.3). For amino acids and organic acids, the ternary component was added in an equimolar ratio relative to the CD concentration, while polymers were added at a fixed concentration of 0.1% (*w*/*v*). All other conditions, sampling steps, and analytical procedures were identical to those used for the binary systems.

### 2.3. Isothermal Stress Tests

To evaluate the compatibility of ALA with potential ternary components meant to improve CD complexation, an isothermal stress test was conducted. The study included monitoring the intrinsic stability of ALA alone and in binary mixtures with selected components, including amino acids (glycine, cysteine, proline, arginine, lysine, aspartic acid, glutamic acid), organic acids (citric acid, gluconic acid, tartaric acid, lactic acid, malic acid, succinic acid), organic base (diethanolamine), and polymers (polyvinylpyrrolidone-K30, chitosan, HP-methylcellulose, hyaluronic acid, polyethylene glycol, poloxamer). For each mixture, about 5 mg of ALA and 5 mg of the ternary component were precisely weighed into 1.5 mL Eppendorf tubes and homogenized to create uniform physical mixtures. ALA alone was weighed similarly to serve as a reference control for inherent thermal stability. All samples were stored at 50 °C in a Memmert UN55 universal oven (Memmert GmbH + Co. KG, Schwabach, Germany) for 3 weeks. Afterward, samples were dissolved in a 1:1 volumetric ratio of methanol and water (HPLC-grade), sonicated, and filtered for HPLC-DAD analysis.

### 2.4. Computational Methods

A combined quantum mechanics (QM) and molecular dynamics (MD) approach was employed to characterize the inclusion behavior, stability, and interaction profiles of α-lipoic acid (ALA) with native cyclodextrins (αCD, βCD, γCD) and their hydroxypropylated derivatives (HPαCD, HPβCD, HPγCD) in aqueous solution. This multi-scenario simulation framework enabled systematic evaluation of inclusion stability, release mechanisms, spontaneous complex formation, and the influence of cyclodextrin size and substitution on ALA solubility and stability under physiologically relevant conditions.

All analyzed structures were drawn in MarvinSketch, version 19.21.7 [52] (Certara, Budapest, Hungary), while all MD simulations were conducted using GROMACS, version 2023.1.2 [53]. Simulations were performed exclusively using the biologically active R-(+)-enantiomer of ALA. All host–guest systems were constructed in a 1:1 molar ratio (ALA/CD). Two protonation states of ALA were considered: protonated and deprotonated, to capture pH-dependent properties relevant to physiological and gastrointestinal conditions.

Initial geometries of ALA were optimized using the B3LYP/6-31+G(d) level of theory with implicit solvation (CPCM, water) using the Gaussian 16 program [54]. Frequency calculations were performed to confirm the absence of imaginary frequencies. Electrostatic potential-derived charges were subsequently computed at the HF/6-31G(d) level using the CHELPG scheme with implicit solvent (CPCM, water), and RESP-compatible charges were derived from these calculations.

Initial geometries of CDs and ALA/CD inclusion complexes were prepared in the VMD program, version 1.9.4a51 [55], prior to MD simulations. Inclusion complexes were manually generated by positioning ALA within the CD cavity to obtain plausible host–guest conformations. These pre-assembled complexes were used to evaluate encapsulation stability and ALA release performance. In a second set of simulations, ALA and CDs were initially placed as physically separated components within the simulation box to model spontaneous association processes under conditions relevant to the gastrointestinal system, thereby probing factors influencing ALA solubility and stability.

Both ALA and CDs were parameterized using the General AMBER Force Field (GAFF) via the Antechamber [56], ensuring internal consistency of bonded and non-bonded interactions across all systems. Each system was placed within an approximately 5 Å octahedral simulation box filled with TIP3P water molecules. Counterions were added as required to ensure overall charge neutrality. Energy minimization was performed using the steepest descent algorithm with a convergence criterion of 1000 kJ mol^−1^ nm^−1^. Initial equilibration was conducted in two phases. An NVT ensemble was first applied using the V-rescale thermostat to maintain temperature at 300 K. This was followed by NPT equilibration using the V-rescale thermostat in combination with the Berendsen barostat to stabilize pressure at 1 bar. Production MD simulations were subsequently carried out in the NPT ensemble using the V-rescale thermostat and the c-rescale barostat to ensure correct sampling of the isothermal–isobaric ensemble. The Verlet cutoff scheme was used for short-range interactions. All simulations were performed with periodic boundary conditions using a timestep of 2 fs. Bond constraints involving hydrogen atoms were applied using the LINCS algorithm. Long-range electrostatic interactions were treated using the particle-mesh Ewald (PME) method, while van der Waals interactions were handled using a cutoff scheme. Production simulations were run for 600 ns for each system. In total, 14.4 μs of production MD simulations were generated across all studied systems. All simulations were performed on the Supek supercomputer at the University of Zagreb University Computing Centre SRCE, using one NVIDIA A100 GPU (40 GB HBM2e memory) and 16 CPU cores based on AMD EPYC 7763 processors per simulation run. The Supek GPU nodes are equipped with four NVIDIA A100 GPUs and a 64-core AMD EPYC 7763 processor, providing the computational resources required for the simulations performed in this study.

Binding free energies and interaction profiles between ALA and CDs were estimated using the Molecular Mechanics Poisson–Boltzmann Surface Area (MM-PBSA) method, as implemented in the gmx_MMPBSA package [57]. Calculations were performed on representative snapshots extracted from equilibrated portions of the trajectories. It should be noted that MM-PBSA calculations provide approximate estimates of binding free energies and are most reliable for comparing relative interaction strengths among structurally related systems. Consequently, the calculated Δ*G*_int_ values were interpreted primarily in a comparative manner to assess trends in host–guest interactions rather than as absolute thermodynamic binding free energies.

This computational protocol closely follows the methodology recently applied in the investigation of dasatinib–CD inclusion complexes, where analogous MD simulations combined with MM-PBSA binding free energy calculations successfully rationalized the stability and release behavior of complexes with native and modified CDs [35]. This consistency enables direct comparison between the two systems and further validates the robustness of the applied approach for studying drug–CD interactions relevant to formulation development.

### 2.5. Preparation of CD Complexes

Grinding method. CD complexes with ALA were prepared using equimolar ALA-to-CD ratios in a high-energy vibrational mill (Mixer Mill MM 500 control, Retch GmbH, Haan, Germany) equipped with 50 mL grinding stations made of ZrO_2_. The batch size (total powder mass) was 1 g, corresponding to 0.13 g of ALA and 0.87 g of HPβCD. The grinding was performed at 30 Hz for 10 min using four ZrO_2_ grinding balls, each 10 mm in diameter. During the grinding, the system was thermostated at 20 °C.

Lyophilization. CDs were dissolved in 50 mL of water at room temperature. The obtained CD solution was heated to 40 °C under magnetic stirring. Once the temperature reached 40 °C, ALA was added, and the solution was stirred for an additional 10 min until complete dissolution, after which it was transferred to a Petri plate. The batch size was 1 g, with an equimolar ALA-to-CD ratio. The freeze-drying protocol included initial sample freezing at −40 °C for 2 h, 22 h of main drying at −20 °C and 0.5 mbar vacuum, and 4 h of secondary drying at 20 °C and 0.0047 mbar, performed using Martin Christ, Epsilon 2–4 LSC plus freeze drier (Osterode am Harz, Germany).

Spray-drying. The aqueous ALA–CD solution prepared as described above was spray-dried using a Büchi B-290 Mini Spray Dryer (Flawil, Switzerland), equipped with a standard 0.5 mm two-fluid nozzle and a high-performance cyclone. Inlet drying air temperature was 120 °C, sample flow rate was set to 4.35 mL/min, pump to 8%, and aspiration was to 100%, resulting in an outlet temperature of 54 °C. Samples were prepared in two batches of 3 g. All batches of solid-state complexes were collected in glass vials and stored in a freezer at −20 °C, protected from the light.

### 2.6. Differential Scanning Calorimetry (DSC)

For DSC analysis (Discovery DSC 250 Differential Scanning Calorimeter, TA Instruments, New Castle, DE, USA), 2–3 mg samples were accurately weighed into aluminum pans, sealed with pierced lids, and scanned under nitrogen purge of 50 mL/min over the temperature range of 10–100 °C, employing a heating rate of 10 °C/min.

### 2.7. X-Ray Powder Diffraction (XRPD)

XRPD analysis was performed using a Panalytical Empyrean diffractometer with Bragg–Brentano geometry (Malvern Panalytical, Worcestershire UK), equipped with a copper anode (CuKα; *λ* = 1.5406 Å), a Ni filter, and a PIXcel3D-Medipix3 detector. Data were collected in continuous scan mode over the 2θ range of 3–40°, with a scan rate of 0.022° s^−1^ and a counting time of 18.87 s.

### 2.8. Fourier-Transformed Infrared Spectroscopy (FTIR)

FTIR analysis was performed on Thermo Scientific™ Nicolet™ Summit X FTIR Spectrometer BGA2410260 (Waltham, MA, USA) over the 4000–400 cm^−1^ range at 2 cm^−1^ resolution. The number of scans per sample was 16, recorded under ambient conditions using an ATR probe. To reduce random noise and improve spectral shape, the Savitzky–Golay algorithm was applied.

### 2.9. Powder Flow Properties

Powder flow properties were determined indirectly using a modified method previously described by our group [58], employing a 5 mL polypropylene syringe as the measuring vessel. Briefly, 500 mg of the powder sample was gently poured into the syringe barrel with the plunger removed, avoiding any intentional compaction. The initial powder volume was recorded as the bulk volume (*V*_0_). The syringe was then tapped manually approximately 100 times, or until no further change in powder volume occurred. The final volume was recorded as the tapped volume (*V*_f_). Bulk density (*ρ*_0_) and tapped density (*ρ*_f_) were calculated using Equations (2) and (3):

(2) $$\rho_{0}=\frac{m}{V_{0}}$$

(3) $$\rho_{f}=\frac{m}{V_{f}}$$

where m is the sample mass. Flow indices (i.e., Hausner ratio and compressibility index) were then derived from the bulk and tapped densities (Equations (4) and (5)):


(4) $$Hauser\;ratio=\frac{\rho_{f}}{\rho_{0}}$$

(5) $$Compressibility\;index\;\left(\%\right)=\frac{\rho_{0}-\rho_{f}}{\rho_{0}}\times 100$$

### 2.10. Moisture Content

The moisture content of the starting compounds and prepared complexes was determined by thermogravimetric analysis (TGA) using a Discovery TGA 550 thermogravimeter (TA Instruments, New Castle, DE, USA). Loss on drying was determined by applying a heating step in the temperature range of 20–105 °C at a heating rate of 10 °C/min, followed by an isothermal step at 105 °C for 15 min.

### 2.11. In Vitro Dissolution Testing

In vitro dissolution testing was performed according to the pH transition procedure described in the European Pharmacopoeia 11.0, monograph 5.17.1 [59]. The study was conducted in triplicate using a scaled-down USP II paddle apparatus (Agilent 708-DS, Agilent Technologies, Santa Clara, CA, USA) with 100 mL of the dissolution media maintained at 37 °C and stirred at 100 rpm.

The initial dissolution medium (pH 1.5) was prepared by dissolving 4.0 g of NaCl in water, then adding 63.2 mL of 1 M HCl, and finally diluting the solution to a total volume of 2000.0 mL. pH transition was achieved by the stepwise addition of a tris(hydroxymethyl)aminomethane (TRIS)/Na-acetate buffer mixture, prepared by dissolving 2.28 g of TRIS and 1.77 g anhydrous Na-acetate in the pH 1.5 medium. The buffer mixture was added at 31 min to adjust the medium to pH 4.5 and again at 46 min to reach pH 7.4, following the procedure described in the pharmacopoeia [59]. The ALA quantity used in the test was 60 mg (free or as prepared CD complexes), corresponding to the standard oral dose of 600 mg in 1 L of the dissolution media.

Aliquots of 3 mL were withdrawn at 5, 10, 15, 30, 35, 45, 50, 55, 60, and 75 min and immediately replaced with an equal volume of pre-warmed medium adjusted to the corresponding pH. Samples were filtered and analyzed by HPLC-DAD at 215 nm to quantify ALA. The percentage of dissolved ALA was calculated by Equation (6):

(6) $$\%\mathrm{Dissolved}=\frac{\mathrm{Mass}\;\mathrm{dissolved}}{\mathrm{Initial}\;\mathrm{dose}}\times 100$$

The obtained in vitro dissolution profiles were compared by calculating a model-independent and widely accepted similarity factor *f*_2_ according to Equation (7):

(7) $$f_{2}=50\times {log}_{10}\left\lfloor \frac{100}{\sqrt[{2}]{1+\frac{\sum_{t=1}^{n}{\left(R_{t}-T_{t}\right)}^{2}}{n}}}\right\rfloor$$

where *n* is the number of time points sampled, *t* is the specific sample time point, *R*_t_ is the mean percentage of the reference drug dissolved at time t, and *T*_t_ is the mean percentage of the test drug dissolved at time *t* [60].

### 2.12. In Vitro Permeability Studies

Permeapad^®^. In vitro permeability studies were conducted using Phoenix dry heat vertical diffusion cells (Teledyne Labs, Chatsworth, CA, USA) with a donor compartment volume of 1.5 mL, acceptor compartment volume of 15 mL, and an effective diffusion surface area of 1.77 cm^2^. Tested samples (free ALA, equimolar physical mixture (HPβ + ALA), and prepared complexes) were dissolved in phosphate buffer solution (PBS, pH 6.8) prepared by mixing 77 mL of 0.2 M NaOH with 6.8 g KH_2_PO_4_ and diluting to 1 L to simulate jejunal conditions. The ALA concentrations in all the samples were 1.0 mM.

The donor compartments of diffusion cells were filled with 1.5 mL of each test solution, while the acceptor compartments contained 15 mL PBS pH 7.4, prepared by dissolving 2.98 g Na_2_HPO_4_·2H_2_O, 0.19 g KH_2_PO_4_, and 8.0 g NaCl in water, then diluted to 1 L. A PermeaPad^®^ GIT Barrier (Innome GmbH, Espelkamp, Germany) was placed between compartments to simulate a passive transcellular barrier. The system was thermostated at 37 °C. At defined time points of 0.5, 1, 1.5, 2, 3, 4, and 5 h, 400 μL were withdrawn from the acceptor compartment to HPLC vials and immediately replaced with an equal volume of the same medium (PBS, pH 7.4). ALA content in the collected samples was analyzed by HPLC-DAD at 215 nm after dilution with 400 μL PBS (pH 7.4). Permeability profiles were constructed by plotting the amount of permeated drug per unit surface area (*dQ*/*A*) as a function of time (*t*). The apparent permeability coefficient (*P_app_*) was calculated according to Equation (8):

(8) $$Papp\;=\frac{dQ}{dt}\;\times \;\frac{1}{A\;\times \;c_{0}}$$

*c*_0_—the initial concentration of the drug in the donor compartment.

Caco-2 monolayer. Human adenocarcinoma Caco-2 intestinal epithelial cells obtained from American Type Culture Collection (ATCC) were grown in DMEM, supplemented with 10% fetal bovine serum at 37 °C in 5% CO_2_ atmosphere [61]. Culture medium was replaced every 2–3 days. The viability of Caco-2 cells was determined using the MTT assay. After being seeded in 96-well plates (Thermo Fisher Scientific 130188, Rochester, NY, USA), cells were grown for 48 h until they reached confluence. After DMEM was aspirated, 90 µL of fresh medium was added with 10 µL of testing samples, the increasing concentrations of ALA (0.25–4 mg/mL), CD–ALA inclusion complexes, and HPβ + ALA (2–32 mg/mL), each in quadruplicate. Samples were dissolved in ethanol, forming stock solutions, then diluted 10–160-fold in Hank’s Balanced Salt Solution (HBSS, pH 7.4); the final ethanol concentration in samples did not exceed 1%. For a positive control, cells were treated with HBSS. Plates were incubated for 2 h at 37 °C, after which cells were washed with PBS, and the 40 µL MTT solution was added to each well at a final concentration of 0.5 mg/mL. After an additional 3 h incubation, 170 µL DMSO was added, the plates were shaken for 30 min, and then absorbance (A) was measured at 570 nm (SepctraMax, Molecular Devices, San Jose, CA, USA). For blank comparison, absorbance was measured in wells containing MTT without cells. Cell viability was expressed as a percentage (%) relative to the positive control according to Equation (9):

(9) $$\%viability=\frac{Asample\;-\;Ablank}{Acontrol\;-\;Ablank\;}\times 100$$

For permeability studies, cell monolayers were grown on a Falcon^®^ 12-well Clear Multiwell Plate comprised of polystyrene with a cell growth area of 3.8 cm^2^. Caco-2 monolayers were used for experiments after 21 days of initial seeding, when the transepithelial electrical resistance (TEER) reached approximately 450 Ωcm^2^. On a day of testing, medium was aspirated from both apical and basolateral chambers and washed with HBSS. Then, 0.4 mL of the sample dissolved in HBSS to correspond to 1 mM ALA was added to the donor compartment of each well. The receiving compartment contained 1.2 mL of HBSS. Samples were incubated at 37 °C and 100 rpm for 2 h in a shaker. At defined points of 0, 0.5, 1, 1.5, and 2 h, 400 µL was withdrawn from the basolateral side of the cell layers and immediately replaced with 400 µL of HBSS, pre-incubated at 37 °C. Collected samples were filtered and analyzed by HPLC-DAD.

### 2.13. Stability Studies

Accelerated stability studies. To conduct an accelerated stability study according to the International Council for Harmonisation of Technical Requirements for Pharmaceuticals for Human Use (ICH) Q1A(R2) recommendations [62], samples prepared in triplicate were stored in a Memmert ICH100L eco stability chamber (Memmert, Schwabach, Germany) at 40 °C and 75% relative humidity, collected and analyzed after 0, 1, 2, 3 and 6 months. Before HPLC-DAD analysis, each sample was dissolved in a 1:1 (*v*/*v*) methanol/water (HPLC-grade) mixture, sonicated, and filtered.

Photostability. Photostability studies were conducted in accordance with ICH Q1B guidelines [63]. For each sample, test units and corresponding light-protected controls (wrapped in aluminium foil) were prepared in triplicate, placed in the Memmert ICH110L eco stability chamber (Schwabach, Germany), and exposed to a combined daylight (8.5 kLux) and UV (5 W m^−2^) light source to achieve a cumulative irradiation of 1,200,000 Lux·h for visible light and 200 W·h·m^−2^ for UV radiation. At the end of the exposure cycle, samples were collected, dissolved in a 1:1 a 1:1 (*v*/*v*) methanol/water (HPLC-grade) mixture, sonicated, and filtered for HPLC-DAD analysis.

Gastrointestinal stability. In vitro simulation of gastrointestinal digestion and the assessment of gastrointestinal stability of selected samples were conducted following the standardized static INFOGEST 2.0 protocol for food, using 1.25× SGF and 1.25× SIF stocks prepared as previously described [50]. ALA stock solution, 1 mg/mL in SGF, was prepared by dissolving 20 mg ALA in 4 mL methanol and diluting to 20 mL with SGF. For the initial concentration sample of ALA, 2 mL of stock solution was diluted to 4 mL with SGF, filtered, and analyzed via HPLC-DAD.

The same sample preparation principle was applied to the other samples analyzed. Analyses were conducted in triplicate. For gastric digestion simulation, 4 mL of the stock solution for each tested sample was added, along with 200 µL pepsin (20 mg/mL). Samples were incubated at 37 °C for 2 h with shaking. After 2 h, to simulate the intestinal phase of digestion, 2 mL SIF, 1.25 mL pancreatin (133 mg/mL), and 0.75 mL porcine bile extract (200 mg/mL) were added. Samples were prepared for analysis by centrifuging for 10 min/20,000× *g* at 4 °C. Supernatant was collected, filtered, and analyzed by HPLC-DAD. Gastrointestinal stability of ALA and samples corrected to ALA were calculated as Equation (10):

(10) $$ALA\;retention\;\left(\%\right)=\frac{ALA\;concentration\;after\;digestion}{ALA\;initial\;concentration}\times \;100$$

Initial concentrations of 980–1000 mg/L of ALA were used in experiments since this range aligns with typical ALA supplement dosages (300–600 mg) dispersed in gastrointestinal fluids.

### 2.14. Chromatographic Determination of ALA

Chromatographic separation was carried out using an Agilent 1260 Infinity II HPLC-DAD system that included an autosampler, controller, pump, degasser, column oven, and diode array detector (Agilent Technologies, Santa Clara, CA, USA). The column used was Kinetex^®^ C8 (250 × 4.6 mm, 5 μm; Phenomenex, Torrance, CA, USA; cat. no. 00G-4608-E0). The mobile phase flow rate was 1.2 mL/min, with the column maintained at 35 °C. Column pressure ranged from 220 to 250 bar, with detection at 215 nm, an injection volume of 20 μL, a retention time of 4.8 min, and a total run time of 6 min. The mobile phase comprised methanol, 0.005 M KH_2_PO_4_, and acetonitrile (1160:920:180, *v*/*v*/*v*), prepared following the protocol described previously [51]. It was adjusted to pH 3.0–3.1 with phosphoric acid, vacuum-filtered through 0.45 µm cellulose acetate membranes (Whatman), and degassed before use. The stock solution was prepared by dissolving 20.1 mg of ALA in 20 mL of methanol/water (1:1, *v*/*v*), initially dissolving in 10 mL of methanol, then adding 10 mL of water. Working standards were created by serial dilution in 10 mL volumetric flasks using the same procedure with methanol/water (1:1, *v*/*v*). All standards were sonicated in an ultrasonic bath and filtered through a FilterBio PES Syringe Filter (25 mm, 0.22 μm) (Labtex Ltd.) into HPLC vials prior to analysis. Chromatographic data acquisition, integration, and analysis were performed with OpenLab CDS software ver. 3.5 (Agilent Technologies, Santa Clara, CA, USA), integrated with the Agilent 1260 Infinity II HPLC-DAD system.

### 2.15. Statistical Analysis

Statistical analysis of permeability and stability data was performed using one-way ANOVA followed by Tukey’s multiple comparison test in GraphPad Prism 10.0 (GraphPad Software, Inc., San Diego, CA, USA). Differences were considered statistically significant at *p* < 0.05.

## 3. Results and Discussion

### 3.1. ALA–CD Inclusion Complexation in the Solution

#### 3.1.1. Selection of CDs—Phase Solubility Studies

**Figure 1:**
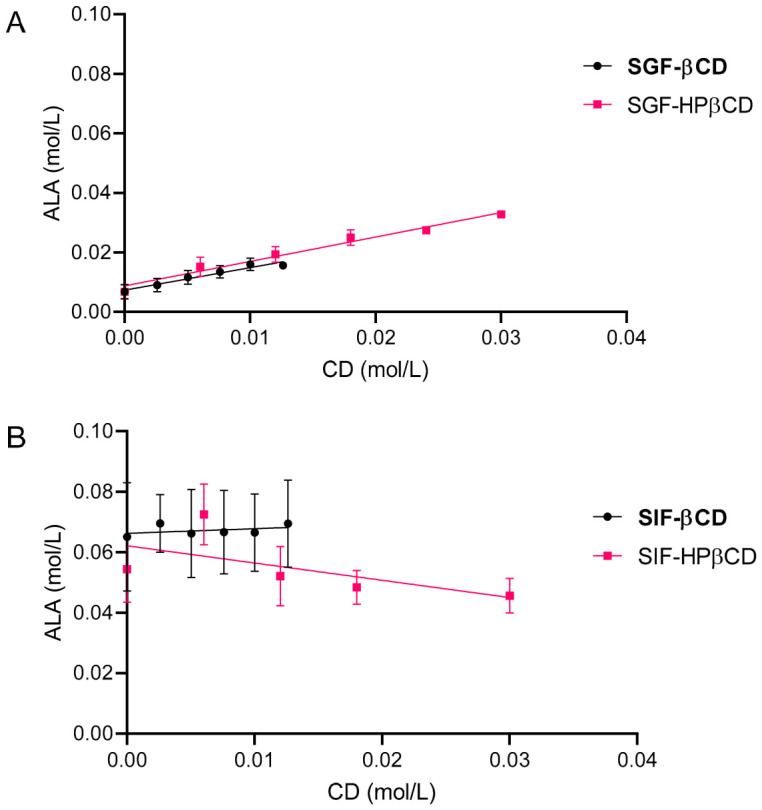
Phase solubility diagrams of ALA with βCD and HPβCD in simulated gastric fluid (SGF, pH = 3.0) (**A**) and simulated intestinal fluid (SIF, pH = 7.0) (**B**).

To gain insights into CD complexation of ALA under physiological conditions, phase solubility studies were performed in SGF (pH 3) and SIF (pH 7.0), prepared without enzymes. Representative phase solubility diagrams for complexes with βCD and HPβCD are presented in Figure 1, while those for the other complexes are given in the Supplementary datasheet (Figure S1). The corresponding parameters obtained by the analysis of the phase solubility diagrams are given in Table 2. ALA is a weak acid with a pKa ranging from 4.76 to 5.4 [64]. Therefore, in SGF, the protonated form of ALA predominates, whereas in SIF, the unprotonated form will be prevalent. Deprotonation of ALA strongly increases the aqueous solubility of ALA (Table 1) and affects its interaction with CDs. In fact, phase solubility curves of ALA with parent CDs in SIF presented a slope near zero, indicating no interaction with the selected CDs in SIF. Such a result aligns with those reported by Takahashi et al. obtained in unbuffered aqueous solutions of αCD, βCD, and γCD, where the unprotonated form is also prevalent [40]. In SGF, different results were obtained. For αCD and γCD, a BS-type phase solubility curve was observed, indicative of the formation of complexes with limited solubility [3], while βCD exhibited an AL-type phase solubility diagram, indicating the formation of soluble inclusion complexes of 1:1 stoichiometry. To the best of our knowledge, previous studies have not systematically investigated the CD complexation of ALA using phase solubility analysis under controlled pH conditions, making it challenging to directly compare the obtained stability constants. Only Maeda et al. [39] reported a Ks value for ALA–βCD complexes of 600 M^−1^, determined in a 40 mM acetic acid buffer of pH 4, where the unprotonated ALA form also prevails.

For hydroxypropylated CDs (HPαCD, HPβCD, and HPγCD, respectively), somewhat different results were obtained (Figure 1 and Figure S1). Again, in SIF, those modified CDs did not enhance the solubility of ALA, whereas in SGF, a linear increase in ALA solubility was observed, corresponding to the AL type of the phase solubility diagram, with the stability constant values following the trend HPβCD >> HPαCD > HPγCD. A higher complex stability constant for HPβCD than for βCD was also reported by Trentin et al. using capillary zone electrophoresis [65]. This observation is consistent with molecular modeling results (Section 3.1.2), which indicate that hydroxypropyl substitution enhances favorable host–guest interactions while maintaining a suitable release profile.

#### 3.1.2. Selection of CDs—In Silico Modeling

Molecular modeling studies were performed to elucidate the molecular basis of inclusion complex formation between ALA and natural as well as HP-substituted CDs, and to rationalize the experimentally observed trends in solubility enhancement and drug release. All simulations were conducted using the biologically active R-(+)-ALA enantiomer in a 1:1 host–guest ratio. Considering the p*K*_a_ of ALA (4.7), both the anionic form (ALA^−^), predominant under intestinal conditions (pH ≈ 7.4), and the neutral form (ALA), relevant for acidic gastric environments, were explicitly investigated. It should be noted that the applied atomistic models represent simplified host–guest systems in aqueous solution and therefore do not fully reproduce the complexity of the experimental conditions, using racemic ALA mixtures and simultaneous presence of several different solubilization mechanisms. Consequently, the simulations are primarily intended to provide mechanistic insight into dominant molecular interactions and dynamic complexation behavior rather than direct quantitative reproduction of all experimental observations.

Molecular dynamics simulations in water were performed in two ways. The first set of simulations was initiated from the manually formed inclusion complexes to inspect their persistence and stability. At the same time, in order to estimate the tendency of ALA to depart the inclusion complex, a process essential for the drug release, another set of simulations was initiated from well-separated components that allowed only surface interactions. A graphical representation of initial structures is presented in Figure S2.

Our analysis revealed that spontaneous formation of inclusion complexes from initially separated components is limited within the simulated timescale (600 ns), with interactions predominantly occurring at the cyclodextrin surface (Figure S3). This is supported by the relatively large average center-of-mass (COM) distances between ALA^−^ and the cyclodextrin cavity centers, which are 12.4 Å, 17.4 Å, and 10.5 Å for αCD, βCD, and γCD, respectively. Nevertheless, all natural CDs exhibited a certain degree of molecular recognition toward ALA^−^ form, following the trend γCD > αCD > βCD, as consistently evidenced by interaction energies (−8.5, −7.4, and −3.7 kcal mol^−1^, in the same order) and center-of-mass (COM) distances. We note in passing that MM–PBSA-derived interaction energies were used primarily to compare the relative stability of different ALA–cyclodextrin systems and binding modes. Therefore, emphasis was placed on energetic trends and differences between systems rather than on the absolute magnitude of the calculated free energies.

In contrast, simulations initiated from pre-formed inclusion complexes showed markedly different stability profiles. Complexes with αCD and βCD remained stable throughout the simulation, whereas γCD failed to maintain a stable inclusion geometry (Figure S4) as indicated by a rather similar COM value and practically unchanged Δ*G*_int_ value relative to simulations initiated from separated components. The calculated interaction energies confirmed significantly stronger binding for αCD (−19.4 kcal mol^−1^) and βCD (−17.0 kcal mol^−1^) compared to γCD (−8.4 kcal mol^−1^). These findings identify αCD and βCD as promising excipients for improving the solubility of ALA and indicate that γCD is unlikely to form stable inclusion complexes in intestinal conditions, which is only partially consistent with the experimental data obtained (Table 2). Given the lack of CD solubilization enhancement observed experimentally in SIF, it can be concluded that ALA solubilization is primarily driven by pH-dependent ionization, while interactions with CDs have an insignificant effect.

**Figure 2:**
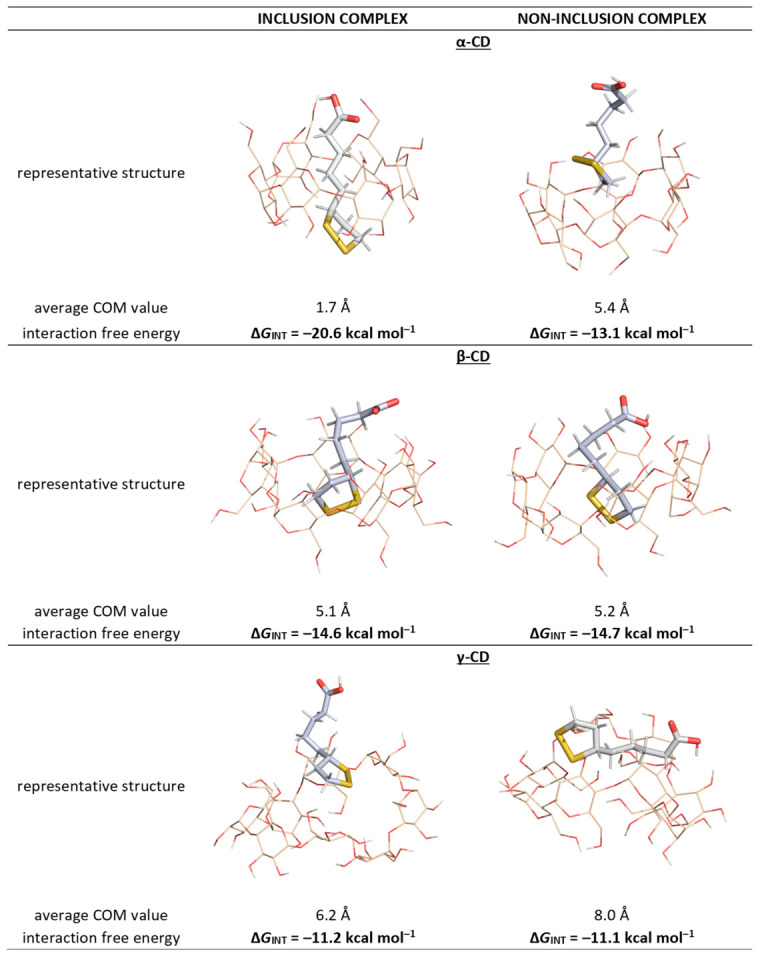
Representative structures of complexes between neutral ALA form and natural cyclodextrins under acidic gastric conditions following 600 ns of molecular dynamics simulations initiated either from manually formed inclusion complexes or well-separated components. Data also include the average center-of-mass (COM) distances between compounds together with the interaction free energies as obtained with the MM-PBSA analysis.

**Figure 3:**
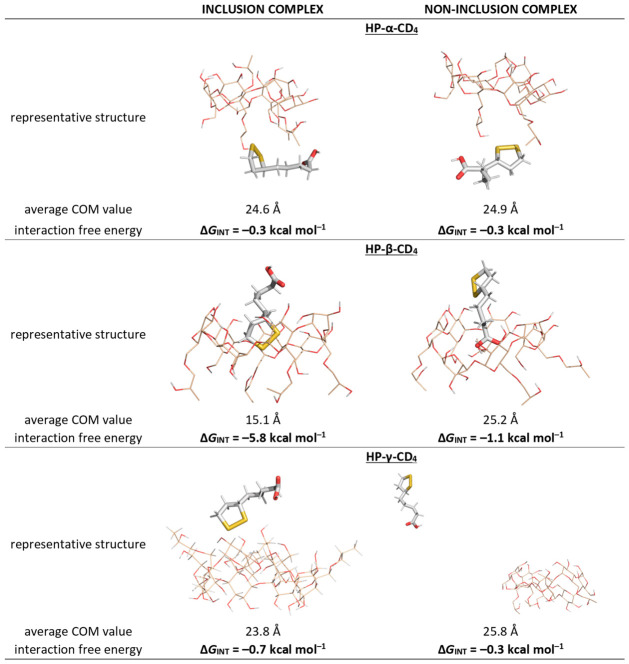
Representative structures of complexes between neutral ALA form and tetrasubstituted HP-CDs under acidic gastric conditions following 600 ns of molecular dynamics simulations initiated either from manually formed inclusion complexes or well-separated components. Data also include the average center-of-mass (COM) distances between compounds together with the interaction free energies as obtained with the MM-PBSA analysis.

Having established the behavior of the anionic ALA^−^ form under intestinal conditions, we next examined the neutral ALA form, which predominates under acidic gastric conditions. The corresponding results for natural CDs are presented in Figure 2, while results for HP-substituted CDs are shown in Figure 3.

To investigate interactions in gastric conditions, analogous simulations were performed for the neutral ALA form. The results revealed a pronounced pH-dependent performance (Figure 2). For βCD, acidification of the medium induces a pronounced change in inclusion complex behavior. While the anionic ALA^−^ forms relatively stable inclusion complexes under intestinal conditions (Δ*G*_int_ = −17.0 kcal mol^−1^), conversion to the neutral form at lower pH leads to a pronounced shift in the nature of host–guest interactions and a reduced preference for persistent inclusion geometries. Notably, the interaction energies of inclusion and non-inclusion configurations become nearly identical (approximately −14.7 kcal mol^−1^), accompanied by increased center-of-mass distances indicative of increased configurational flexibility and transient displacement of ALA from deeply included geometries. This suggests that, under acidic conditions, ALA no longer exhibits a strong energetic preference for deeply included geometries within the βCD cavity, while still maintaining dynamic host–guest contacts, including partially surface-associated configurations. From a biopharmaceutical perspective, these findings suggest that βCD maintains dynamic host–guest interactions under acidic conditions, while exhibiting reduced stabilization of persistent inclusion geometries. Such behavior may facilitate a balance between transient complexation, solubility enhancement, and subsequent ALA release during gastrointestinal transit.

In contrast, αCD exhibits a more balanced yet still strongly stabilizing response to pH changes. Under physiological conditions, inclusion complexes with ALA^−^ are highly stable (Δ*G*_int_ = −19.4 kcal mol^−1^), and this stability is further enhanced upon acidification (Δ*G*_int_ = −20.6 kcal mol^−1^ for neutral ALA). At the same time, the energetic difference between inclusion and non-inclusion states decreases under acidic conditions, indicating a partial shift toward a more dynamic equilibrium. However, the remaining stability gap (ΔΔ*G*_int_ = −7.5 kcal mol^−1^) still reflects a pronounced thermodynamic preference for the inclusion geometry. This suggests that, despite some weakening of the relative stabilization, ALA remains substantially retained within the αCD cavity in gastric conditions. In spite of the energetically favorable interaction, the obtained experimental data showed formation of ɑCD complexes with limited solubility, diminishing their biopharmaceutical significance (Figure S1A).

In the case of γCD, no significant stabilization of inclusion complexes was observed upon acidification, which is consistent with experimental data (Figure S1A). Both protonation states of ALA resulted in similar interaction energies for inclusion and non-inclusion configurations (approximately −11 kcal mol^−1^), accompanied by relatively large COM distances, indicating a lack of stable encapsulation. Furthermore, simulations initiated from pre-formed inclusion complexes frequently evolved toward non-inclusion arrangements, confirming the intrinsic instability of the host–guest system. Taken together, these findings indicate that none of the native cyclodextrins achieve an optimal balance between complex stability and pH-responsive drug release, thereby underscoring the need for chemically modified derivatives, such as HP-substituted CDs, with improved performance.

Modeling HP-substituted CDs is challenging due to their intrinsic structural heterogeneity. Commercial HP-CDs are not single-defined compounds but complex mixtures differing in both the degree and positional distribution of substitution on the glucose units [66]. This variability, combined with the large number of possible substitution sites, leads to a combinatorial space of isomers that cannot be fully represented in a single atomistic model. Therefore, simulations rely on simplified representative structures with defined substitution patterns. Previous computational studies have addressed this issue by employing models with fixed and limited degrees of substitution to approximate experimentally relevant systems. Such approaches use discrete substitution patterns to investigate structure–property relationships, assuming that dominant physicochemical trends are still captured despite the underlying heterogeneity [67,68]. It has also been shown that, while substitution degree can influence thermodynamic properties, complexation performance is often more strongly governed by host–guest interactions than by the exact substitution number [69].

Accordingly, we adopted a pragmatic modeling strategy using tetrasubstituted HP-CDs (HPαCD_4_, HPβCD_4_, and HPγCD_4_) as representative systems. This provides a balance between structural realism and computational feasibility, in line with prior literature using partially substituted models. The chosen substitution pattern also maximizes spatial separation between hydroxypropyl groups, reducing artificial steric crowding and offering a more realistic description of substituted cyclodextrin cavities.

Under intestinal conditions (anionic ALA^−^ form), HPβCD_4_ demonstrated markedly superior complexation behavior compared to its α- and γ-counterparts (Figures S5 and S6). Simulations initiated from well-separated components showed spontaneous formation of a stable inclusion complex, evidenced by a low average COM distance (4.5 Å) and a highly exergonic interaction free energy (Δ*G*_int_ = −20.4 kcal mol^−1^). Even when starting from the pre-formed inclusion geometry, the complex remained stable with a favorable Δ*G*_int_ value of −4.6 kcal mol^−1^. In contrast, HPαCD_4_ and HPγCD_4_ exhibited weaker and more dynamic surface-associated interactions with ALA^−^, characterized by large COM distances and near-zero interaction energies (Δ*G*_int_ ≈ −1 to −2.4 kcal mol^−1^), indicating negligible encapsulation under these conditions, consistent with the limited solubility enhancement observed experimentally for these derivatives.

The behavior under acidic gastrointestinal conditions (neutral ALA form) is presented in Figure 3. HPβCD_4_ retained its ability to form a stable inclusion complex with neutral ALA, displaying a clearly favorable interaction free energy for the inclusion geometry (Δ*G*_int_ = −5.8 kcal mol^−1^) relative to the non-inclusion configuration (Δ*G*_int_ = −1.1 kcal mol^−1^), together with a more compact COM distance. This indicates that HPβCD_4_ effectively encapsulates the lipophilic dithiolane and alkyl chain moieties of neutral ALA even at low pH. However, the interaction energy with ALA is significantly lower than with ALA^−^ (Δ*G*_int_ = −14.6 kcal mol^−1^), which may facilitate efficient drug release while preserving favorable host–guest association once the encapsulated formulation reaches the gastrointestinal tract. In contrast, both HPαCD_4_ and HPγCD_4_ did not show a pronounced energetic preference for persistent inclusion geometries of the neutral form, with interaction energies of the inclusion and non-inclusion states being nearly identical (approximately −0.3 kcal mol^−1^ for HPαCD_4_ and −0.7 vs. −0.3 kcal mol^−1^ for HPγCD_4_). These are accompanied by large COM distances indicative of predominantly surface or solvent-exposed interactions rather than true cavity inclusion. These findings suggest negligible host–guest recognition for the neutral form of ALA with HPαCD_4_ and HPγCD_4_. These observations are consistent with the obtained experimental data (Table 2, Figure S1).

Taken together, the molecular dynamics simulations provide a clear molecular rationale for the experimental phase solubility results, in which HPβCD emerged as the optimal host with the highest apparent stability constant (*K*_s_ = 668.9 ± 10.6 M^−1^) in simulated gastric fluid.

#### 3.1.3. Selection of Ternary Compounds

**Figure 4:**
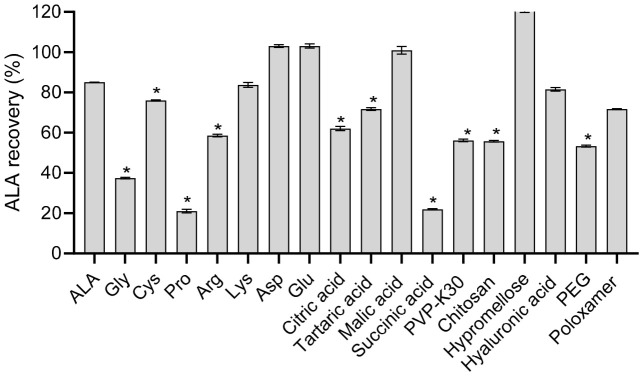
Compatibility of binary mixtures of ALA and selected ternary components (at 1:1 mass ratio) in an isothermal stress test performed at 50 °C for three weeks. Samples marked with an asterisk (*) presented a significantly higher degree of ALA degradation in the tested binary mixtures (*p* < 0.05) compared to that of pure ALA. Abbreviations: Gly—glycine; Cys—cysteine; Pro—proline; Arg—arginine; Lys—lysine; Asp—aspartic acid; Glu—glutamic acid; PVP-K30—polyvinylpyrrolidone K30; PEG—polyethylene glycol; ALA—α-lipoic acid.

The addition of small amounts of various additives, such as amino acids, organic acids and bases, and water-soluble polymers, in aqueous complexation media can increase the complexation efficiency of CDs. This permits the use of considerably lower quantities of CDs, thereby optimizing the cost, safety profile, and formulation volume in the final product [70,71]. Based on that, we have considered several amino acids, organic acids, organic bases, and polymers to further enhance the complexation and solubilization efficiency of HPβCD for ALA. Preference was given to structurally and functionally diverse candidates representing different interaction mechanisms (e.g., ionic interactions, hydrogen bonding, steric effects, etc.). The first step in this phase of the research involved a compatibility study of ALA and selected additives, since their compatibility with HPβCD had already been established in previous research [70]. The compatibility studies were performed by an isothermal stress test coupled with HPLC quantification of ALA. Although time-consuming, this approach is widely used, offering the most direct and reliable method for assessing drug–excipient compatibility [71]. The remaining ALA quantities in the equimolar mixtures after 3 weeks of storage at 50 °C, expressed as the recovery relative to the initial content in the sample, are presented in Figure 4. Under these conditions, pure ALA degraded approximately 15%. Consequently, any additive that resulted in a markedly higher degradation level was excluded from further investigation.

Based on the results of the isothermal compatibility testing, phase solubility studies in SGF were performed using Lys, Asp, Glu, and malic acid, applied in equimolar amounts with respect to HPβCD, while polymers (hypromellose and hyaluronic acid) were added to the complexation medium in a concentration of 0.1% (*w*/*w*). The stability constant values obtained from the phase solubility studies in SGF are presented in Table 3.

In all ternary systems tested, the obtained *K*s values were significantly lower than those for the ALA–HPβCD binary system, indicating the lack of the tested ternary compounds to further enhance the solubilization potential of HPβCD. In fact, in some cases, it was even decreased. The largest decrease in *K*s value, reflecting significantly lower ALA solubilization, was observed for Asp and Mal, whereas the smallest was observed for hypromellose. In SGF, both side-chain (pKa~2.1) and α-carboxyl group (pKa~9.8) of Asp are protonated, as well as the amino group (pKa~9.6), giving Asp a positive net charge. Therefore, positively charged Asp is unlikely to compete strongly with ALA for HPβCD cavities. In ternary systems, Asp may influence complexation indirectly by altering microenvironmental pH, hydrogen bonding networks, and CD hydration [72] or by interacting with ALA via hydrogen bonding. The negative effects of the other amino acids tested may be explained by a similar mechanism. On the other hand, malic acid (pKa1~3.4 and pKa2~5.1) has a neutral net charge in SGF [73], which may allow its inclusion into the central cavity of HPβCD [74], thereby competitively displacing ALA. Hypromellose is a neutral polymer with a high molecular weight that interacts with HPβCD by non-inclusion complex formation, thus having the least pronounced effect on the ALA complexation. Given the lack of additional ALA solubilization in the ternary systems examined, further studies were limited to ALA–HPβCD binary systems. It should be noted, however, that this decision was based solely on phase solubility analysis, and that potential benefits of ternary additives beyond equilibrium solubility (as improved wettability or supersaturation maintenance) were not evaluated and cannot be excluded.

### 3.2. Selection of Inclusion Complex Preparation Technique—Solid State Characterization

Selection of the technique for preparing inclusion complexes in the solid state is a crucial step that influences the physicochemical properties and performance of the final product and must be carefully tailored to the characteristics of both the CD and the drug, as well as the intended purpose of CD complexation [75]. In this study, we considered grinding in a high-energy vibrational mill as a versatile, highly efficient, fast, and sustainable solvent-free technique for preparing CD complexes in the solid state (HPβALA-gr), aligning with the shift toward technologies consistent with green chemistry principles [76,77]. However, as the potential of grinding for production at the industrial level is still evolving, we have also considered spray-drying as a fast, continuous, and highly efficient method that is frequently used in the pharmaceutical and food industries to produce various products with enhanced functionality and stability [78] and has not been investigated yet for drying ALA–CD complexes. Finally, lyophilization as a CD complex production technique was also considered, as it provides very mild drying conditions, suitable for thermo-labile molecules such as ALA [79]. Based on the results of the first phase of this research, we have focused our interest on ALA complexes with HPβCD. However, none of the employed techniques enabled the preparation of ALA samples without HPβCD, which is required to evaluate the effect of the preparation technique on its physicochemical properties. High-energy milling led to pronounced adhesion and compaction of pure ALA on the vessel walls, preventing its effective processing. The limited aqueous solubility of ALA did not allow the preparation of solutions concentrated enough for either spray-drying or freeze-drying. Additionally, the low melting point of ALA causes it to adhere to heated parts of the spray-drying apparatus, preventing recovery of the dry product.

**Figure 5:**
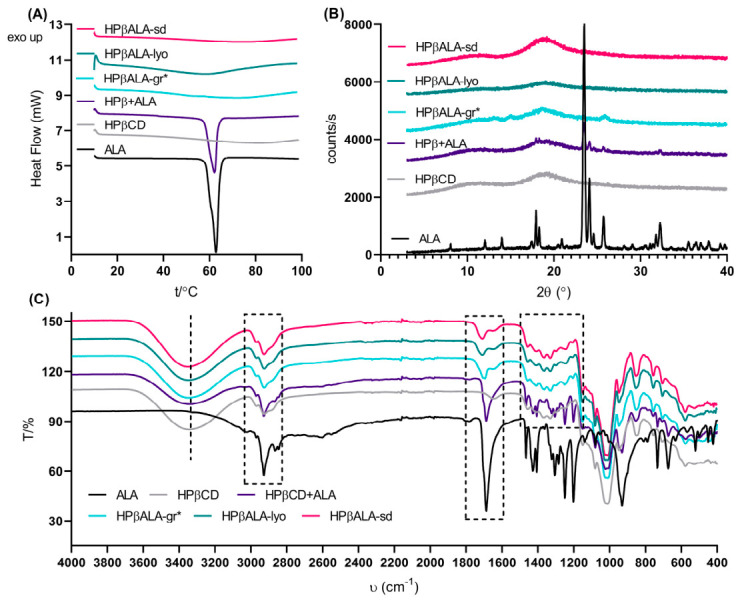
Solid-state characterization of starting compounds (ALA and HPβCD), HPβ + ALA, and complexes prepared by grinding (HPβALA-gr), lyophilization (HPβALA-lyo), and spray-drying (HPβALA-sd) performed by DSC (**A**), XRPD (**B**), and ATR-FTIR (**C**). Asterisk (*) denotes the sample where significant ALA degradation was observed.

The ALA–CD inclusion complexes prepared by grinding (HPβALA-gr), lyophilization (HPβALA-lyo), and spray-drying (HPβALA-sd) were analyzed using DSC coupled with XRPD to monitor the solid-phase transitions of ALA, while FTIR was used to investigate the chemistry of interactions occurring in the prepared samples [75]. However, no single analytical technique, nor even a combination of techniques, can unequivocally confirm inclusion complex formation in the solid state. The results should therefore be interpreted as indirect, supportive evidence of intermolecular interactions rather than definitive proof of host–guest inclusion. DSC thermogram of pure ALA (Figure 5A) presented a sharp endothermic peak with onset and peak temperatures of 59.62 °C and 62.78 °C, corresponding to its melting with a fusion enthalpy (Δ*H*_fus_) of 119.48 J/g. This is consistent with the results of Mendoza et al. [80], reporting an onset and peak temperature of 61.26 °C and 64.09 °C, respectively, with a Δ*H*_fus_ of 95.77 J/g. On the contrary, Racz et al. [42] reported a melting temperature of 66 °C and fusion enthalpy of 208 J/g, whereas Ikuta et al. [20] reported a fusion temperature of 50 °C for the R-isomer of ALA, without providing the Δ*H*_fus_ value.

XRPD of ALA presented several intense diffraction peaks at 2Θ° values of 23.49, 24.12, and 25.78, aligned with previous reports [80], and confirming the crystalline nature of this compound, consistent with DSC data. The polymorphism of ALA is not reported.

In the examined temperature range, the DSC thermogram of HPβCD presented only a wide endothermic peak corresponding to evaporation of adsorbed moisture, while XRPD revealed a halo effect, typical for an amorphous compound (Figure 5A,B). In the DSC thermogram of the HPβ + ALA, the intensity of the ALA fusion peak is reduced proportionally to its content in the analyzed sample, while the XRPD pattern of this sample represents the superimposition of the spectra of pure compounds. All this indicates a lack of any solid-state interaction in the HPβ + ALA. In the DSC thermograms of all prepared complexes (Figure 5A), the ALA fusion peak was not observed, while the XRPD diffractograms of HPβALA-gr showed only a few low-intensity peaks, the most prominent of which appeared at 25.78° 2Θ. According to the XRPD data, HPβALA-lyo and HPβALA-sd complexes are completely amorphous (Figure 5B). Such disappearance of crystallinity and formation of amorphous systems is commonly interpreted as indicative of strong drug–CD interactions; however, it may arise from multiple phenomena and does not, by itself, provide unequivocal evidence of inclusion complex formation in the solid state [75]. However, we have demonstrated in our previous investigations that when such an amorphous product is dissolved in water, it is instantly transformed into an inclusion complex [81]. Therefore, the present findings should be more appropriately interpreted as evidence of amorphous, interacting systems capable of forming inclusion complexes upon dissolution.

Further insight into the chemistry of the interaction between ALA and HPβCD causing the observed amorphization was obtained by ATR-FTIR spectroscopy (Figure 5C). In the spectrum of ALA, the most prominent absorption bands appeared in the carbonyl stretching region (1687.3 cm^−1^), the aliphatic C–H stretching zone (2929.5 cm^−1^), and the fingerprint region associated with C–O, C–C, and S–S vibrational modes (1200–1300 cm^−1^) [80]. Likewise, HPβCD exhibited intense O–H stretching (3200–3600 cm^−1^), C–H stretching near 2920–2900 cm^−1^, and characteristic C–O–C vibrations between 900 and 1200 cm^−1^ [42].

The spectrum HPβ + ALA represented a simple superimposition of the spectra of pure components, where all typical ALA and HPβCD bands retained their usual positions, with intensity reduced proportionally to the content of each compound in the mixture. The spectra of the prepared complexes showed distinct differences compared with those of the HPβ + ALA. In the O–H stretching region, complexes presented less intense and slightly narrower bands, suggesting an increase in intermolecular hydrogen bonding among the CD hydroxyl groups caused by complexation [43]. Furthermore, in the ATR-FTIR spectra of the complexes, the C=O stretching band of ALA underwent distinct shifts compared to the HPβ + ALA, appearing at 1703–1715 cm^−1^. Such displacement reflects changes in the polarity and hydrogen-bonding environment around the carbonyl group, which are consistent with drug–CD interactions, potentially associated with partial inclusion or close association within the CD structure arising from CD encapsulation [42,43]. The reduction in intensity of the aliphatic C–H stretching bands of ALA in the 2800–3000 cm^−1^ region may suggest the insertion of the alkyl chain of ALA into the HPβCD central cavity, resulting in altered vibrational dynamics [82]. Additionally, in the 1200–1300 cm^−1^ region, containing C–O and S–S/C–S vibration bands of ALA, a significant reduction in intensity was observed with respect to the HPβ + ALA, indicating that encapsulation affects not only the polar groups but also regions of the ALA backbone containing the disulfide functionality. Taken together, these findings support the presence of significant intermolecular interactions between ALA and HPβCD, which are consistent with, but do not conclusively prove, the formation of an inclusion complex.

The computational insights (Section 3.1.2). also corroborate the ATR-FTIR characterization data. The favorable inclusion geometries identified for HP-β-CD, involving hydrophobic contacts with the dithiolane ring and alkyl chain of ALA together with hydrogen-bonding opportunities near the cavity rim, are consistent with the observed shifts in the C=O stretching band (to 1703–1715 cm^−1^) and the attenuation of aliphatic C–H and S–S/C–S vibrational bands in the ATR-FTIR spectra of the HP-β-ALA complexes. However, it should be noted that molecular modeling provides theoretical support for favorable host–guest interactions and possible binding modes, but does not constitute direct experimental evidence of inclusion in the solid state. Furthermore, the partial dynamic character of the HP-β-CD–ALA complex under acidic conditions supports the excellent dissolution performance, while the fact that significant portions of the ALA molecule remain partially solvent-exposed and accessible to light and oxygen explains why photostability was not markedly improved by complexation.

Further physicochemical characterization of the prepared products focused on determining the bulk and tapped densities, which were used to calculate the compressibility index and Hauser ratio, indicators of powder flow characteristics. Powder flow is a crucial factor in several pharmaceutical manufacturing processes, as insufficient flow reduces process efficiency and leads to suboptimal product quality [83]. The results obtained are presented in Table 4. Both HPβALA-gr and HPβALA-sd have bulk densities comparable to that of ALA, whereas the value for HPβALA-lyo was significantly lower, making it highly voluminous, as is typical of products obtained by lyophilization [79]. After tapping, the powders showed lower density, consistent with their high compressibility index (Table 4). For HPβALA-gr and HPβALA-lyo, the compressibility index and Hauser ratio values indicate poor flow properties, whereas for HPβALA-sd, very poor flow properties are likely. However, this can be further improved by using appropriate glidants and other processing measures [83].

Moisture content is another important product quality attribute, influencing powder flowability and chemical stability, among other properties [84]. In this study, moisture content was determined gravimetrically using TGA (i.e., loss on drying). Since the prepared HPβALA complexes contain no other volatile components or residual solvents, the observed loss on drying is assumed to correspond to the moisture content. ALA, as a hydrophobic compound, has very low moisture content (Table 4). Among the prepared products, HPβALA-gr contains the highest moisture level because grinding was performed under ambient conditions (RH 60–70%) without an additional drying step. Moreover, the HPβCD used in the preparation contains a similar moisture level (i.e., 5.84 ± 0.07%). Both HPβALA-lyo and HPβALA-sd contained somewhat lower moisture content with respect to that of HPβALA-gr (Table 4), indicating that appropriate processing parameters for each drying method were applied. The effect of moisture on the chemical stability of ALA in the selected product was evaluated through accelerated stability studies, presented later in the text.

Different preparation techniques impose diverse types and levels of stress on the processed compounds, potentially affecting their stability during manufacturing [78,79,85]. Therefore, the ALA content of the prepared complexes was quantified and expressed as a recovery relative to the initial ALA quantity used in the preparation (Table 4). For HPβALA-gr, the highest reduction in ALA recovery was observed, indicating that approximately 40% of ALA degraded during grinding. Although the grinding process was carried out for 20 min while the treated components were thermostated at 20 °C, local heat transfer during collisions between the treated material and the grinding balls cannot be excluded. Since mechanochemical processing involves high energy transfer by inducing shear stress, compression, and impact on the treated material, it may promote phase transitions, mechanochemical activation, and potential degradation [86]. In this sense, mechanochemical complex preparation by grinding was excluded from further investigation. On the other hand, spray-drying, despite imposing substantial thermal and mass-transfer stresses on the treated material, did not lead to notable ALA degradation (Table 4). This can be attributed to the very short residence time of droplets in the drying chamber, typically only a few seconds, which limits thermal exposure. Moreover, the rapid solvent evaporation produces pronounced cooling, resulting in particle temperatures significantly lower than the inlet air temperature [78]. This evaporative cooling effect creates a more favorable thermal environment during drying and likely contributes to preserving ALA’s chemical stability during processing. Surprisingly, HPβALA-lyo exhibited an apparent ALA recovery exceeding 100% (Table 4). This overestimation may result from several factors, including a lower residual moisture content than in the starting materials, especially HPβCD, and potential matrix-related analytical enhancement of the ALA signal [87]. In addition, weighing inaccuracies associated with handling the highly porous, low-density lyophilizate may further contribute to the elevated recovery values.

**Figure 6:**
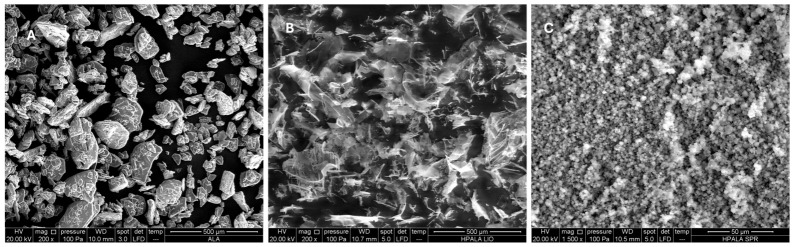
SEM micrographs of ALA (**A**) and HPβCD complexes prepared by lyophilization (HPβALA-lyo; (**B**)), and spray-drying (HPβALA-sd; (**C**)).

Finally, the morphology of the HPβALA-lyo and HPβALA-sd complexes was analyzed using SEM and compared to that of pure ALA (Figure 6). ALA appeared as irregular, angular particles with plate-like and fragment-like shapes. The crystals have poorly defined edges, tending toward elongated and shard-like forms rather than well-developed faceted geometries. The surfaces appear relatively smooth, although several particles exhibit fracture planes, indicating brittle mechanical behavior (Figure 6A). HPβALA-lyo displays a highly porous, flaky structure typical of freeze-dried solids. This structure consists of interconnected, irregular porous networks with thin, brittle walls formed by the sublimation of frozen solvent. No distinct crystalline particles are visible; instead, the product appears as lightweight, low-density fragments (Figure 6B). HPβALA-sd appears as small, uniform spherical particles with a relatively narrow size distribution, formed by the drying of atomized droplets.

### 3.3. Comparative Stability of ALA–CD Inclusion Complexes Prepared by Different Preparation Techniques

**Figure 7:**
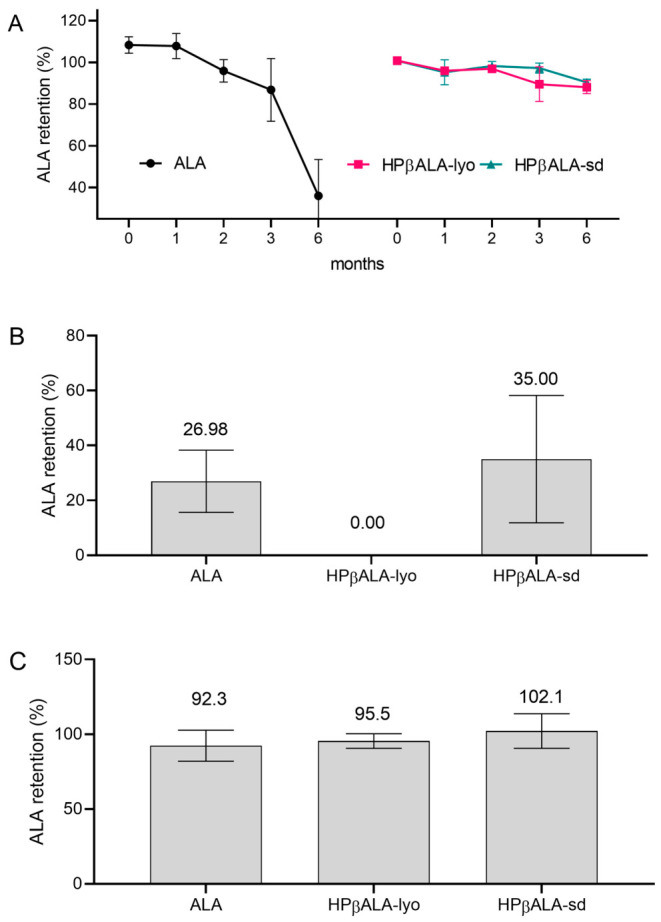
Stability (**A**), UV stability (**B**), and gastrointestinal stability (**C**) of HPβALA prepared by different drying methods (spray-drying and lyophilization). ALA—alpha lipoic acid; HPβALA-lyo—inclusion complex of ALA and HPβCD dried by lyophilization; HPβALA-sd—inclusion complex of ALA and HPβCD dried by spray-drying. % of retention was calculated as the % of the initial amount of ALA determined in the respective treated sample (stored in a climate chamber, UV-treated, or submitted to gastrointestinal digestion).

HPβALA-lyo and HPβALA-sd were compared to ALA in terms of storage stability (Figure 7A), UV stability (Figure 7B), and gastrointestinal stability (Figure 7C) in order to guarantee shelf-life under diverse environmental conditions; investigate possible photodegradation during manufacturing, packaging, and consumer use; and ensure preserving bioactivity through digestion, respectively.

Accelerated stability testing under controlled conditions (ICH-compliant stability chamber at 75% relative humidity with sampling at 0, 1, 2, 3, and 6 months) was chosen in this case because it allows early identification of degradation pathways and instability without waiting for real-time shelf-life data. The described design aligns with the conditions recommended in ICH Q1A(R2) guidelines [62], for accelerated testing of drug substances and products intended for long-term storage at room temperature, thereby supporting registration-ready stability data packages. As presented in Figure 7A, formation of HPβALA-lyo,-sd inclusion complexes significantly improved storage stability of ALA by successfully preventing disintegration during 6-month storage (stability of HPβALA-lyo and HPβALA-sd after 6-month storage was 88.2 ± 3.1% and 90.5 ± 1.5%, respectively). On the contrary, the stability of pure ALA within the same period was only 36.0 ± 17.4%, with the greatest intensity of degradation observed in the period 3–6 months of storage.

To our knowledge, the impact of CD-complexation on the storage stability of ALA has not been investigated so far. Maeda et al. [39] compared the thermal stability of ALA and SBEβALA inclusion complex during a 10 h thermal stress test at 100 °C. They showed that more than 40% of ALA decomposed after 2 h at 100 °C, while the remaining amount of ALA in SBEβALA after 10 h was 79 and 86%, respectively, depending on the method of complex preparation (kneading and freeze-drying). Similarly, Takahashi et al. [40] proved improved thermal stability of mG2βCDALA, βCDALA, ɑCDALA, and γCDALA after 5 h exposure to 100 °C. Ikuta and co-workers [20] proved significantly improved stability of ɑCDALA, βCDALA, and γCDALA compared to ALA under simulation of unfavorable storage conditions (1 h, 2 h, 5 h, 24 h, and 48 h at 25 °C or 70 °C under 100% relative humidity).

ALA is particularly unstable under light exposure, and its photosensitivity stems from its distorted five-membered 1,2-dithiolane ring structure. This leads to decomposition under irradiation and results in diminished physiological activity, necessitating effective light protection strategies [88]. Some advanced encapsulation techniques, such as formulation of nanostructured lipid carriers (NLC), have been proven to significantly improve ALA photostability [88], while other approaches, such as silica-based formulations, did not show a significant positive effect [89].

As presented in Figure 7B and Table S1, exposure to cumulative irradiation of 1,200,000 Lux·h for visible light and 200 W·h·m^−2^ for UV radiation caused significant degradation of both crystalline ALA and HPβALA-sd (65% degradation), while HPβALA-lyo degraded completely, and ALA was not detected after the incubation period. These results show that formulation of ALA–CD inclusion complexes, unlike formulation of NLC, does not improve the photostability of ALA. A possible reason for that is that in CD inclusion complexes, part of the molecule often remains exposed to the environment. Additionally, although CDs provide a molecular-scale cavity for the ALA molecule, they do not form a continuous, dense barrier to dissolved oxygen or diffusing radicals. Consequently, UV-VIS light can still interact with the exposed portion of the chromophore, allowing direct excitation and ring-opening despite the partial inclusion, while photogenerated radicals and oxygen can access ALA relatively freely, promoting chain-type oxidative degradation [20]. Contrary to that, in NLCs, ALA is dissolved or dispersed in a solid–liquid lipid blend that forms a core that acts as a light-scattering and absorbing layer that scatters and partially absorbs photons before they reach the incorporated ALA. Additionally, the lipid core and surfactant shell in NLCs create a diffusion barrier for oxygen and small radicals, slowing the chain-propagation steps in the photodegradation pathway [90].

Interestingly, in HPβALA-lyo, degradation of ALA was complete, indicating that lyophilized ALA–CD complexes are particularly prone to photodegradation. This could be explained by specific morphological characteristics of HPβALA-lyo. Namely, as is obvious from the SEM images (Figure 6) and data presented in Table 4, lyophilized HPβALA had a highly porous, flaky structure, which increases the amount of ALA present at the particle surface and, thus, is exposed to light and oxygen. Additionally, it facilitates rapid diffusion of oxygen and moisture into the bulk, accelerating oxidative pathways during irradiation. On the contrary, crystalline ALA and HPβALA-sd had a dense, compact spherical morphology that shielded ALA from light and air over time [90]. In general, such light-sensitive products should be protected using UV-blocking packaging, opacifying film coatings, and controlled-light manufacturing conditions to prevent photodegradation and ensure product stability [91].

Investigating gastrointestinal stability of ALA is a desirable step in the process of formulation optimization because studies have demonstrated that free R(+)-ALA retains only 43% integrity after 1 h at pH 1.2 and 37 °C, mimicking stomach conditions, probably due to polymerization that involves formation of irreversible covalent linkages forming oligomeric or polymeric species [20]. Ikuta et al. have reported that CD complex formation successfully protected ALA under gastric conditions [20]. Our results show that ALA is stable under gastrointestinal conditions, regardless of formulation, with average gastrointestinal stability ranging from 92.3% (ALA) up to 102.1% (HPβALA-sd) (Figure 7C). The observed differences arise from different experimental conditions applied for the in vitro simulation of gastrointestinal digestion. While Ikuta et al. [20] simulated gastric digestion at pH = 1.2, we used the INFOGEST protocol, which maintains a constant gastric pH of 3.0 during the gastric digestion phase. This value simulates the average pH in the fed stomach state after buffering by a meal, balancing fasted-state acidity (pH 1.3–2.5) with postprandial rises to 5 or higher. It supports reproducible static digestion for 2 h at 37 °C with pepsin (2000 U/mL), aligning with human in vivo endpoints for nutrient breakdown. It stands out among in vitro gastrointestinal digestion models due to its standardization of physiological parameters, enabling reproducible comparisons across studies [50].

### 3.4. Comparative In Vitro Bioavailability of ALA–CD Inclusion Complexes

#### 3.4.1. In Vitro Dissolution Testing

**Figure 8:**
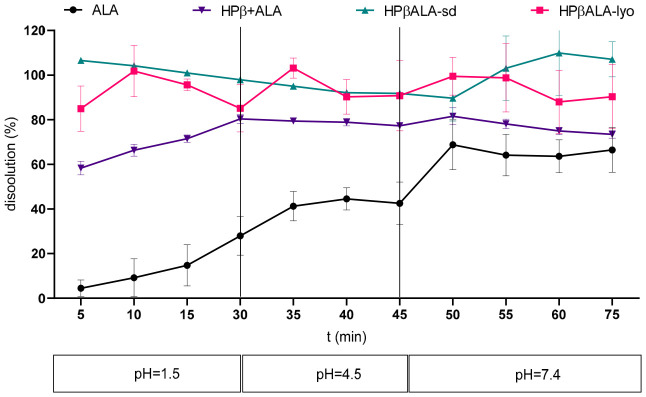
In vitro dissolution profiles of ALA, HPβ + ALA, and complexes of ALA with HPβCD prepared by lyophilization and spray-drying (HPβALA-lyo and HPβALA-sd), under conditions designed to simulate gastrointestinal pH transitions.

In vitro dissolution testing is a key quality-control method for assessing the rate and extent of drug release from a dosage form under standardized conditions. During early pharmaceutical development, it helps optimize formulation and processes by guiding the selection of appropriate dosage forms and excipients to achieve the desired bioavailability and therapeutic effects. Throughout manufacturing and regulatory quality control, dissolution testing plays a vital role in confirming batch-to-batch consistency and ensuring the ongoing stability and quality of the drug product. Additionally, dissolution testing is frequently employed to establish bioequivalence between a drug product and a suitable reference formulation by demonstrating similarity in their dissolution profiles [92]. In this study, in vitro dissolution testing was conducted using a biorelevant dissolution setup designed to mimic gastrointestinal pH transitions, in accordance with the guidelines in monograph 5.17.1 of the European Pharmacopoeia [59]. Under these conditions, the in vitro dissolution properties of pure ALA and prepared CD complexes (i.e., HPβALA-lyo and HPβALA-sd) were compared to assess potential enhancement of ALA oral bioavailability, considering the framework of the Biopharmaceutics Classification System (BCS), where drug dissolution and permeability are recognized as key determinants of oral bioavailability. The obtained in vitro dissolution profiles are presented in Figure 8, and the profiles were compared using the model-independent similarity factor *f*_2_, a metric widely accepted by regulatory agencies. The similarity factor *f*_2_ provides a global measure of dissolution profile similarity, with values between 50 and 100 indicating similar profiles while values below 50 indicate dissimilarity (Table 5). This approach is particularly sensitive to differences exceeding approximately 10% at corresponding sampling points [60].

In vitro ALA dissolution in SGF is markedly limited, but increases upon the pH shift due to ALA ionization, reaching approximately 66% of the dose dissolved by the end of the experiment. Addition of HPβCD, even as HPβ + ALA, markedly increased the amount of ALA dissolved, especially during the acidic stage of the dissolution test, likely due to in situ inclusion complex formation. Molecular modeling confirmed that HPβCD maintains sufficient complex stability in acidic media to enhance solubility while allowing dynamic release, consistent with the rapid dissolution profiles observed. Moreover, the in vitro dissolution of HPβ + ALA appears to be pH independent. In fact, differences between dissolution profiles were quantitatively confirmed by similarity factor *f*_2_ analysis, which yielded values below 50 for all pairwise comparisons, indicating significant dissimilarity between dissolution profiles of pure ALA and HPβCD-based systems, as well as between the individual HPβCD-based formulations (Table 5). The dissolution of HPβALA-lyo is completed within the first 5 min of the dissolution test, regardless of the acidic pH of the media. Such behavior may be attributed to the morphology of the lyophilized product, which provides a larger surface area in contact with the dissolution medium, thereby enhancing the dissolution rate alongside the solubilizing effect of HPβCD complexation. However, during the gastric and duodenal phases of the test, a slight decrease in the dissolved fraction was observed, indicating supersaturation, where the dissolved ALA concentration exceeded its thermodynamic solubility at that pH, followed by re-dissolution under conditions simulating the intestinal environment due to ALA ionization. The HPβALA-sd exhibited an in vitro dissolution profile largely comparable to that of the lyophilized product, although with slightly more pronounced fluctuations in the dissolved ALA fraction (Figure 8). The somewhat lower dissolved ALA concentration observed within the first 5 min for HPβALA-sd can be attributed to its distinct particle morphology. Unlike the highly porous, flake-like structure of the lyophilized complex, which provides extensive effective surface area and facilitates immediate contact with the dissolution medium, the spray-dried material consists of compact, spherical particles with a reduced contact surface area. As a result, the initial dissolution rate of HPβALA-sd is briefly lower, but the profiles converge once wetting and particle dispersion become complete.

#### 3.4.2. Permeability Profiling in Caco-2 Monolayers and Permeapad^®^ Biomimetic System

Permeability plays a crucial role in drug absorption and bioavailability following oral dosing. It can be assessed through diverse experimental approaches, from sophisticated in situ techniques like intestinal perfusion systems to simpler in vitro models using cell cultures (e.g., Caco-2 monolayers) and basic cell-free artificial membranes (e.g., PAMPA^®^, Permeapad^®^) [93].

The major goals of our tests were to investigate the impact of ALA–CD inclusion complexation (achieved by different technologies approaches) on the permeability across the Caco-2 monolayer (i), and to assess the applicability of the cell-free permeation model for the assessment of the comparative permeability of ALA from different formulations (ii).

**Figure 9:**
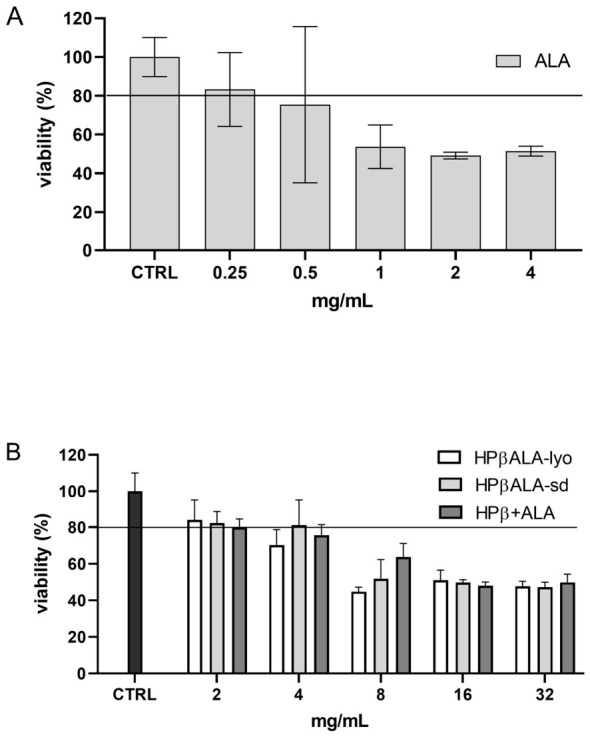
Cytotoxicity of ALA (**A**); inclusion complexes of ALA and HPβCD (HPβALA-lyo and HPβALA-sd) and HPβ + ALA (**B**) as determined by MTT-test. CTRL—negative control (cell media-DMEM); ALA—alpha lipoic acid; HPβALA-lyo—inclusion complex of ALA and HPβCD dried by lyophilization; HPβALA-sd—inclusion complex of ALA and HPβCD dried by spray-drying; HPβ + ALA—physical mixture of ALA and HPβCD.

As described previously, prior to conducting Caco-2 permeability assessment, cytotoxicity using the MTT test was conducted after 2 h exposure of Caco-2 cell monolayers to different concentrations of ALA (0.25–4 mg/mL); HPβALA-lyo, HPβALA-sd, or HPβ + ALA (2–32 mg/mL) (Figure 9). The aim was to identify the highest non-toxic concentration that maintains cell viability above 85–90%, preventing disruptions in tight junction integrity or paracellular leakage, thereby guaranteeing the reliability and reproducibility of apparent permeability coefficient (Papp) measurements. Additionally, post-exposure TEER measurements (>300 Ω·cm^2^) confirmed the maintenance of a polarized epithelial phenotype and barrier function, mitigating false positives arising from compromised monolayers.

The results indicate that 0.25 mg/mL (1.2 mM) ALA and 4 mg/mL HPβALA-lyo, HPβALA-sd, or HPβ + ALA represent the highest concentrations that maintain 80–100% viability of the cell monolayer after 2 h of exposure. Based on these findings, 1 mM ALA was selected for all subsequent permeability experiments. To achieve this ALA concentration consistently, 0.20 mg/mL ALA and 1.65 mg/mL HPβALA-lyo, HPβALA-sd, or HPβ + ALA were employed in every assay, all corresponding to the 1 mM ALA.

**Figure 10:**
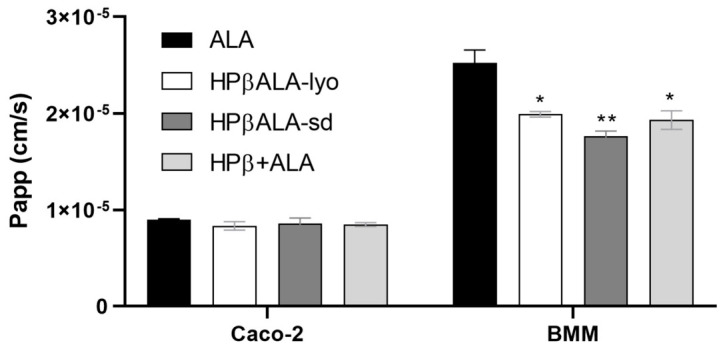
Papp values of ALA, CD-inclusion complexes, and HPβ + ALA determined in the Caco-2 model and by using biomimetic membranes. ALA—alpha lipoic acid; HPβALA-lyo—inclusion complex of ALA and HPβCD prepared by lyophilization; HPβALA-sd—inclusion complex of ALA and HPβCD prepared by spray-drying; HPβ + ALA—physical mixture of ALA and HPβCD; * values are statistically different compared to ALA (*p* < 0.001); ** values are statistically different compared to ALA *p* < 0.0001.

Results of permeability testing (Figure 10) showed that Papp ranged from 8.32 × 10^−6^ cm/s (HPβALA-lyo) up to 8.99 × 10^−6^ cm/s (ALA) with no statistically significant differences among tested samples. These results show that the presence of CDs or incorporation of ALA into CD-based inclusion complexes does not affect its permeability and that, based on the obtained Papp values all tested formulations can be categorized with medium permeability (1–10 × 10^−6^ cm/s), which corresponds to 20–70% absorbed fraction in vivo [94] and is consistent with available in vivo data for ALA [1,17,18,19].

The obtained Papp values were somewhat lower than those reported by Uchida et al. [95], who assessed Caco-2 permeability of the R-(+) and S-(−) enantiomers of ALA. In their study, Papp ranged from 14.3 × 10^−6^ to 16.7 × 10^−6^ cm/s, depending on the enantiomer and initial ALA concentration in the apical chamber (20–500 µg/mL), indicating high absorption (>70%).

Observed differences likely stem from the significantly higher initial ALA concentrations used by Uchida et al. (20–500 µg/mL vs. 20 µg/mL in our study) [95]. Higher concentrations can yield apparently elevated Papp values due to two major mechanisms: (i) existence of saturable transporters or efflux pumps, or (ii) toxicity effects of the investigated substance on the Caco-2 cell monolayer. The literature evidence [96] supports the involvement of the monocarboxylate transporter in ALA uptake, which is consistent with mechanism (i). Mechanism (ii) was confirmed in this work, as ALA concentrations > 25 µg/mL exhibited toxicity, potentially disrupting tight junction integrity or promoting paracellular leakage (as shown in Figure 9A). Notably, Uchida et al. selected ALA concentrations based on solubility studies rather than toxicity assessments, whereas TEER (the primary indicator of monolayer integrity) was measured only prior to permeability experiments [95].

To our knowledge, the applicability of cell-free artificial membranes (e.g., PAMPA^®^, Permeapad^®^) for the investigation of ALA permeability has not been investigated so far. The non-cellular models offer key benefits in terms of speed and affordability, which is particularly valuable in the initial development stages, when screening permeability for numerous candidates demands high efficiency. The main disadvantage of cell-free permeation systems is that they only predict passive diffusion of drugs; therefore, their applicability depends on the relative contributions of passive and saturable, transport-dependent transport for a particular drug [97]. In this study, Permeapad^®^ artificial membranes were used, since they offer advantages over the predominantly used PAMPA system. The Permeapad^®^ system is ready-to-use, storable at room temperature, exhibits superior long-term integrity and stability across wide pH ranges, and its biomimetic phospholipid structure maintains robustness to solvents and bio-relevant media, supporting combined dissolution–permeation assays without compromising barrier function. Additionally, it has been studied for numerous drug substances and provides excellent correlation with PAMPA and Caco-2 permeability studies [93,98].

As presented in Figure 10, Papp values of investigated samples ranged from 17.6 × 10^−6^ cm/s (HPβALA-sd) up to 25.2 × 10^−6^ cm/s (ALA) and were significantly higher compared to those obtained in the Caco-2 cell monolayer. Additionally, the presence of CD in the solution significantly decreased ALA permeability (*p* < 0.001). This effect was not further investigated, but it may have been caused by CD–ALA complexation, which reduced the fraction of free drug and, consequently, its permeability through PAMPA due to the size/polarity of CD–ALA complexes [99]. Namely, dissolution data (Figure 8) show that at pH 7.4 in the presence of CDs, the concentration of free ALA ranged from 73.44% and higher, meaning that even lower estimates can be expected for the free ALA in the PermeaPad^®^ experiments, which were also conducted at pH 7.4 but omitted the gastric digestion step completely. Additionally, cyclodextrins have been known to interact with phospholipids, cholesterol, and proteins in membranes, altering their fluidity, stability, and permeability [100].

The obtained results are partially expected because Papp values from Caco-2 cell experiments may significantly differ from those from cell-free models owing to the biological complexity of the cellular barrier. The PermeaPad^®^ model exhibits significant limitations when applied to drugs with substantial active transport components, as it is specifically designed to evaluate passive diffusive transport only. Only cell-based systems—depending on the cell type employed—also capture carrier-mediated transport and transcytosis. Consequently, for drugs undergoing active absorption, PermeaPad^®^ may either overestimate or underestimate in vivo absorption, depending on the drug’s affinity for efflux or uptake transporters [98,101].

Caco-2 monolayers express efflux transporters and metabolic enzymes that actively reduce net permeation of many compounds, unlike artificial lipid membranes, which primarily measure passive diffusion. Additionally, non-specific binding to cells or Transwell^®^ surfaces, incomplete recovery due to adsorption, and tighter paracellular junctions in well-differentiated Caco-2 cells contribute to underestimated Papp, particularly for lipophilic drugs [93]. This was confirmed by the study of Jacobsen and co-authors (2020), who compared apparent permeability (P_app_) values of selected drugs estimated using different in vitro models, including artificial membranes (PermeaPad^®^ and PAMPA) and Caco-2 cell cultures [101]. Among the investigated compounds, P_app_ values for acyclovir, enalapril, norfloxacin, sulpiride, and terbutaline were significantly overestimated by PermeaPad^®^ relative to Caco-2-derived values. This finding aligns with the results obtained in our study.

When the goal of a permeability study is to compare different compounds or formulations rather than predict in vivo bioavailability, lower Papp values do not render the model invalid. However, to justify using Permeapad^®^ in place of the Caco-2 model, both should yield consistent trends, which was not observed in our study.

As presented previously, in vitro dissolution testing demonstrated significant enhancement in all ALA–CD inclusion complexes dissolution, with a maximum 94.14% dissolution efficiency of HPβALA-lyo under the intestinal conditions (pH 7.4) compared to 65–74% obtained for ALA. Further investigations in the Caco-2 cell monolayer showed that intestinal permeability was unaffected by the presence of CD in the formulation. Based on the obtained in vitro data, enhanced in vivo absorption of ALA–CD inclusion complexes (compared to ALA) might be assumed. Namely, ALA is typically classified under BCS Class II, where dissolution rate often dictates overall bioavailability [102,103]. ALA–CD inclusion complexes enhance in vitro dissolution efficiency by increasing the drug’s solubility and release kinetics in simulated gastrointestinal fluids, addressing the rate-limiting dissolution step without altering Caco-2 permeability, which remains unchanged and reflects intact membrane transport properties. This aligns with established in vitro correlation principles for BCS Class II compounds, where faster dissolution leads to higher luminal drug concentrations available for absorption, thereby justifying expectations of improved in vivo exposure [103].

## 4. Conclusions

This study established HPβCD complexation of ALA as a transformative formulation strategy to overcome the compound’s inherent biopharmaceutical limitations, including low aqueous solubility and chemical instability. Various ternary systems were tested to achieve additional benefits, but considering the lack of additional ALA solubilization, the study remained limited to ALA–HPβCD binary systems.

Spray-drying and lyophilization emerged as particularly efficacious preparation methods, yielding fully amorphous complexes, as confirmed by complementary thermal, crystallographic, and spectroscopic analyses. Molecular dynamics simulations coupled with MM-PBSA binding free energy calculations provided a clear mechanistic rationale for the superiority of HPβCD, suggesting stable inclusion complex formation with the anionic ALA under physiological conditions while retaining sufficient dynamic character for efficient pH-triggered release of the neutral form in the acidic gastric environment.

ALA–HPβCD complexes demonstrated significant enhancements in dissolution kinetics, achieving supersaturated ALA release (>85% within 5 min) under biorelevant pH-shift conditions (pH 1.5→4.5→7.4), which was in contrast to crystalline free ALA’s protracted profile (~66% at equilibrium). Such rapid and complete dissolution, while preserving medium permeability (Papp 8–9 × 10^−6^ cm/s) across non-cellular (Permeapad^®^) and cellular (Caco-2) barriers, is indicative of sustained transcellular flux and improved bioavailability, which still needs to be confirmed by an in vivo pharmacokinetic study.

Stability profiling of HPβCD inclusion complexes of ALA proved a significant protective effect of CDs—under accelerated ICH conditions (40 °C/75% RH, 6 months), 88–90% HPβCDALA was retained, versus 36% retention of the free drug. On the other hand, the critically low photostability of ALA was not affected by CD complexation, probably due to the chemical characteristics of ALA–CD inclusion complexes that leave significant parts of ALA exposed to UV-VIS light, allowing direct excitation and ring-opening despite the partial inclusion, while photogenerated radicals and oxygen can access ALA relatively freely, promoting chain-type oxidative degradation.

It is important to emphasize that the advancements mentioned above were achieved via scalable and solvent-minimal technologies. Spray-drying offers industrial throughput with favorable powder flow, and lyophilization provides an eco-friendly solid-state alternative. The GRAS status of HPβCD facilitates seamless progression into novel food and nutraceutical formats, circumventing the peroxidation risks and scalability hurdles associated with lipid-based carriers (e.g., SLNs, liposomes).

This work addresses several existing gaps s in ALA–CD inclusion complex research, including underexplored preparation modalities, comprehensive stability under ICH/physiochemical stressors, and integrated bioperformance (dissolution–permeability–stability triad). Future investigations should prioritize in vivo pharmacokinetics in relevant models to quantify absolute bioavailability gains.

## Figures and Tables

**Table 2:** Parameters obtained by phase solubility studies of ALA with different CDs in SGF and SIF.

		SGF			SIF	
	S_0_ (g/L)	Isotherm Type	Ks	S_0_ (g/L)	Isotherm Type	Ks
αCD	1.58 ± 0.28	BS		16.08 ± 5.35	No significant increase in solubility	
HPαCD		AL	343.9 ± 27.7			
βCD		AL	459.2 ± 40.5			
HPβCD		AL	668.9 ± 10.6			
γCD		BS				
HPγCD		AL	134.4 ± 12.7			

S_0_—initial solubility; Ks—apparent stability constant; CD—cyclodextrin; SGF—simulated gastric fluid; SIF—simulated intestinal fluid.

**Table 3:** Parameters obtained by phase solubility studies of ALA with HPβCD in the presence of selected ternary compounds in SGF.

	S_0_ (g/L)	Isotherm Type	Ks (M^−1^)
HPβCD	1.58 ± 0.28	AL	668.9 ± 10.6
HPβCD + Lys			315.5 ± 42.2 **
HPβCD + Asp			236.1 ± 13.3 **
HPβCD + Glu			300.5 ± 30.9 **
HPβCD + Mal			253.8 ± 31.5 **
HPβCD + Hyal			437.2 ± 57.2 *
HPβCD + Hypm			619.4 ± 18.3

S_0_—initial solubility; Ks—apparent stability constant; CD—cyclodextrin; SGF—simulated gastric fluid; SIF—simulated intestinal fluid; Lys—lysine; Asp—aspartic acid; Glu—glutamic acid; Mal—malic acid; Hyal—hyaluronic acid; Hypm—hypromellose; ** values in the column are significantly lower compared to HPβCD (*p* < 0.0001); * values in the column are significantly lower compared to HPβCD (*p* < 0.05).

**Table 4:** Bulk (*ρ*_0_) and tapped density (*ρ*_1_), compressibility index (*CI*) and Hauser ratio (*HR*), moisture content, and ALA recovery for HPβALA-gr, HPβALA-lyo, and HPβALA-sd. Values for pure ALA are presented for comparison.

Sample	*ρ*_0_/(g mL^−1^)	*ρ*_1_/(g mL^−1^)	*CI*/(%)	*HR*	Moisture (%)	ALA Recovery (%)
ALA	0.37 ± 0.01	0.52 ± 0.01	29.1 ± 3.7	1.41 ± 0.08	0.16 ± 0.06	-
HPβALA-gr	0.39 ± 0.01	0.55 ± 0.03	27.7 ± 5.4	1.39 ± 0.10	6.45 ± 0.24 ^b,c^	64.16 ± 3.28 ^a^
HPβALA-lyo	0.03 ± 0.00 ^a^	0.04 ± 0.00 ^a^	28.3 ± 2.9	1.40 ± 0.05	5.08 ± 0.06 ^b,c^	116.33 ± 4.17 ^a^
HPβALA-sd	0.29 ± 0.01	0.42 ± 0.01	32.2 ± 2.6	1.48 ± 0.06	4.68 ± 0.04 ^b^	100.54 ± 4.33 ^a^

^a^—statistically significant difference with respect to the other samples (*p* < 0.0001); ^b^—statistically significant difference compared to ALA; ^c^—statistically significant difference compared to HPβALA-gr (*p* < 0.0001).

**Table 5:** Pairwise similarity factor (*f*_2_) values for ALA and CD-based formulations, including HPβ + ALA, and complexes of ALA with HPβCD prepared by lyophilization and spray-drying (HPβALA-lyo and HPβALA-sd, respectively). The *f*_2_ values ≤ 50 indicate product dissimilarity.

Sample	*Similarity Factor f*_2_ (%)			
	ALA	HPβ + ALA	HPβALA-lyo	HPβALA-sd
ALA	-	20.58	12.00	9.60
HPβ + ALA	20.58	-	34.33	27.65
HPβALA-lyo	12.00	34.33	-	44.77
HPβALA-sd	9.60	27.65	44.77	-

## Data Availability

The original contributions presented in this study are included in the article/Supplementary Materials. Further inquiries can be directed to the corresponding author.

## Supplementary Materials

The following supporting information can be downloaded at https://www.mdpi.com/article/10.3390/pharmaceutics18070780/s1: Figure S1: Phase solubility diagrams of ALA with αCD, γCD, and their hydroxypropylated derivatives (HPαCD and HPγCD, respectively) in simulated gastric fluid (SGF, pH = 3.0) (A) and simulated intestinal fluid (SIF, pH = 7.0) (B); Figure S2: Initial structures for molecular dynamics simulations involving two protonation forms of ALA with natural CDs corresponding to manually formed inclusion geometries and well-separated components. Water solvent molecules are omitted for clarity; Figure S3: Representative structures of complexes between ALA^−^form and natural CDs in intestinal environment following 600 ns of molecular dynamics simulations initiated from the well-separated components. Data also include the evolution of the center-of-mass (COM) distances between components and their average values, together with the interaction free energies as obtained with the MM-PBSA analysis; Figure S4: Representative structures of complexes between ALA^−^form and natural CDs in intestinal environment following 600 ns of molecular dynamics simulations initiated from manually formed inclusion complexes. Data also include the evolution of the center-of-mass (COM) distances between components and their average values, together with the interaction free energies as obtained with the MM-PBSA analysis; Figure S5: Representative structures of complexes between ALA^−^form and tetrasubstituted HP-CDs in intestinal environment following 600 ns of molecular dynamics simulations initiated from the well-separated components. Data also include the evolution of the center-of-mass (COM) distances between components and their average values, together with the interaction free energies as obtained with the MM-PBSA analysis; Figure S6: Representative structures of complexes between ALA^−^form and tetrasubstituted HP-CDs in intestinal environment following 600 ns of molecular dynamics simulations initiated from manually formed inclusion complexes. Data also include the evolution of the center-of-mass (COM) distances between components and their average values, together with the interaction free energies as obtained with the MM-PBSA analysis; Table S1: UV stability of HPβALA prepared by different drying methods (spray-drying and lyophilization).

## Abbreviations

The following abbreviations are used in this manuscript:

ALA	Alpha lipoic acid
BCS	Biopharmaceutical classification system
CD	Cyclodextrin
C_max_	Peak plasma concentration
DSC	Differential Scanning Calorimetry
DMEM	Dulbecco’s Modified Eagle Medium
DMSO	Dimethyl sulfoxide
FTIR	Fourier-Transform Infrared Spectroscopy
HBSS	Hanks’ Balanced Salt Solution
HP	Hydroxypropyl
HPβALA-gr	HPβCD-ALA inclusion complex prepared by grinding
HPβALA-sd	HPβCD-ALA inclusion complex prepared by spray-drying
HPβALA-lyo	HPβCD-ALA inclusion complex prepared by lyophilization
HPβ + ALA	Physical mixture of ALA and HPβCD
HPLC-DAD	High-Performance Liquid Chromatography with Diode Array Detection
ICH	International Council for Harmonisation of Technical Requirements for Pharmaceuticals for Human Use
Ks	Apparent stability constant
MD	Molecular dynamics
MTT	3-(4,5-Dimethylthiazol-2-yl)-2,5-diphenyltetrazolium bromide
Papp	Apparent permeability coefficient
PBS	Phosphate buffer solution
SGF	Simulated gastric fluid
SIF	Simulated intestinal fluid
SLN	Solid lipid nanoparticle
TEER	Transepithelial electrical resistance
TGA	Thermogravimetric analysis
TRIS	Tris(hydroxymethyl)aminomethane
XRPD	X-Ray powder diffraction
