# Supplementary material for: Enhancing the Pharmaceutical Profile of Alpha Lipoic Acid: Cyclodextrin Inclusion Complexation for Improved Stability and Bioavailability
Source: Pharmaceutics. 2026 Jun 25;18(7):780. doi: 10.3390/pharmaceutics18070780 (PMC13416384; doi:10.3390/pharmaceutics18070780)
Supplement: Supplementary file 1 [file pharmaceutics-18-00780-s001.zip › pharmaceutics-4345124-supplementary.pdf]

Supplementary Materials

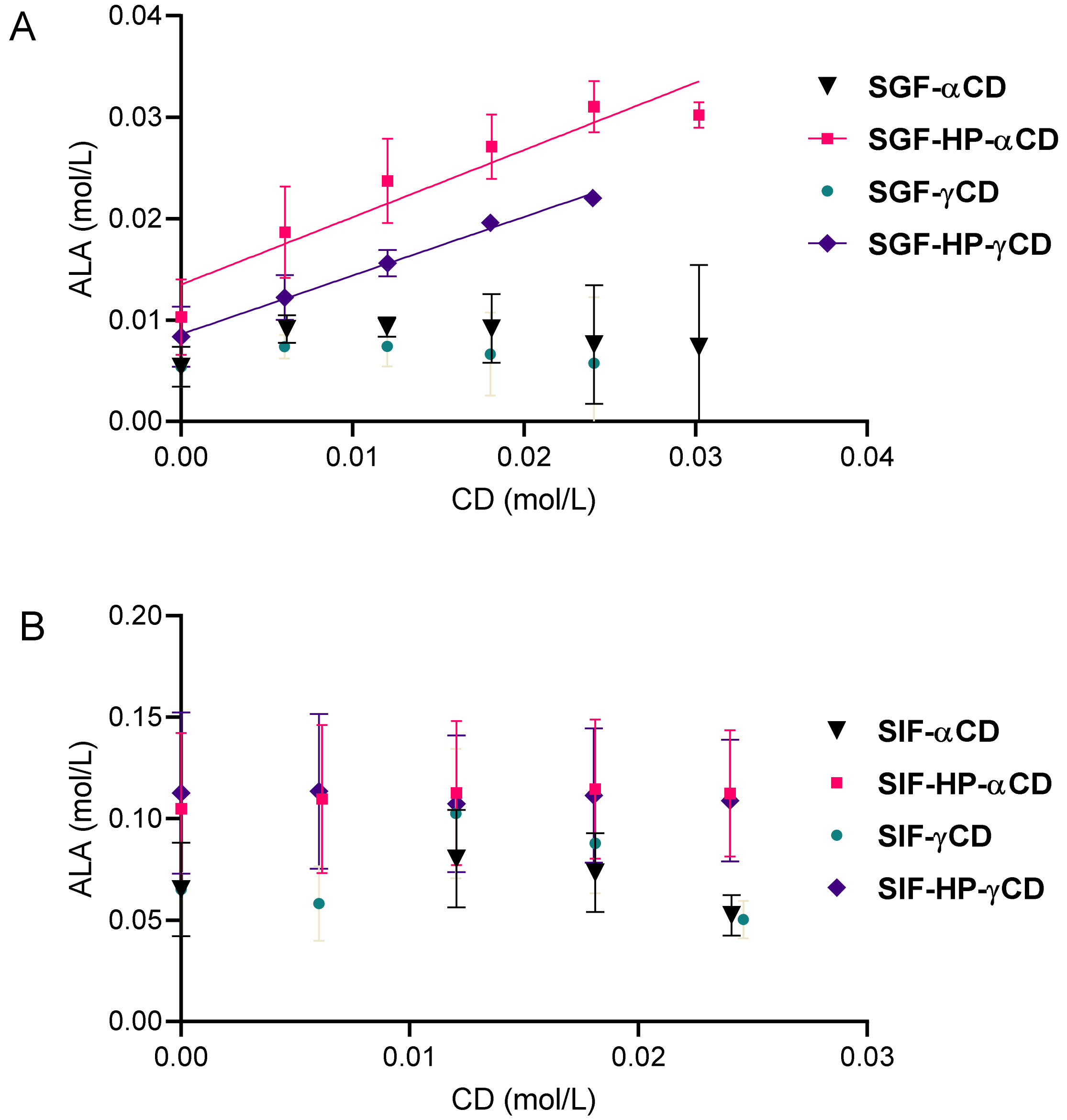

Figure S1. Phase solubility diagrams of ALA with αCD, γCD, and their hydroxypropylated derivatives (HPαCD and HPγCD, respectively) in simulated gastric fluid (SGF, pH=3.0) (A) and simulated intestinal fluid (SIF, pH= 7.0) (B).

Form	Complex type	α-CD	β-CD	γ-CD
**ALA**	inclusion	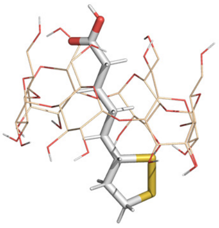	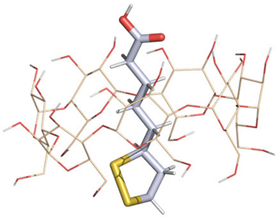	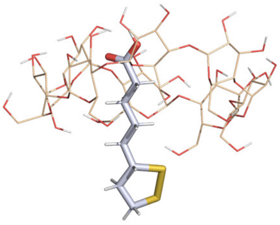
**ALA**	non-inclusion	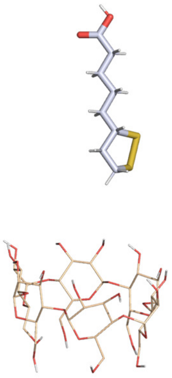	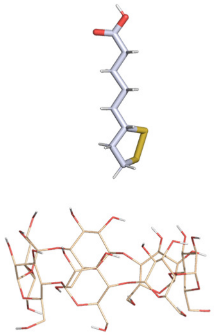	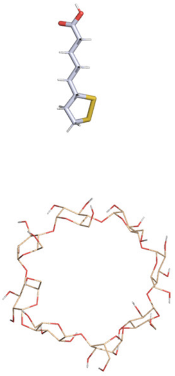

Form	Complex type	α-CD	β-CD	γ-CD
**ALA⁻**	inclusion	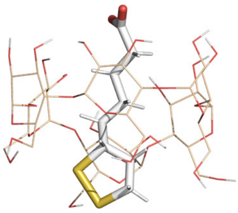	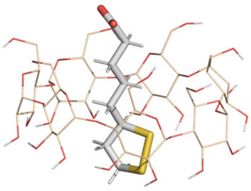	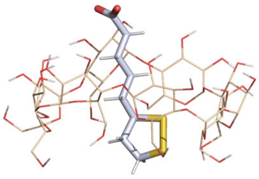
**ALA⁻**	non-inclusion	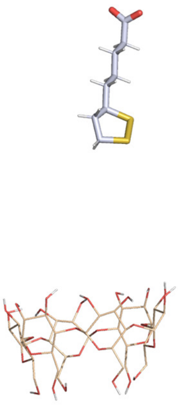	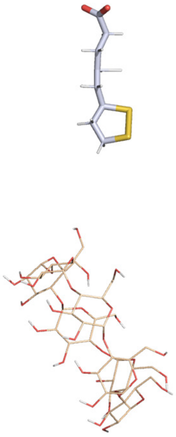	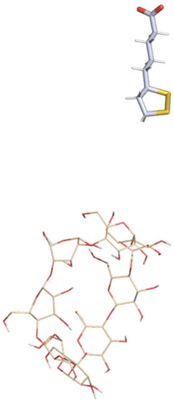

Figure S2. Initial structures for molecular dynamics simulations involving two protonation forms of ALA with natural CDs corresponding to manually formed inclusion geometries and well-separated components. Water solvent molecules are omitted for clarity.

α-CD:ALA$^-$ COM graph

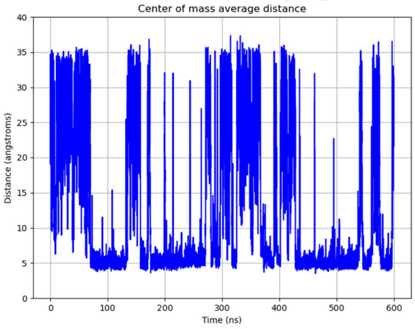

average COM value = 12.4 Å

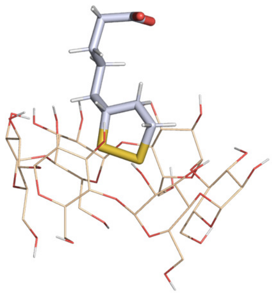

representative α-CD:ALA$^-$ structure

interaction free energy

ΔG_{INT} = –7.4 kcal mol^{-1}

β-CD:ALA$^-$ COM graph

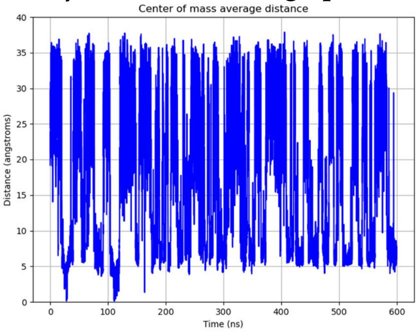

average COM value = 17.4 Å

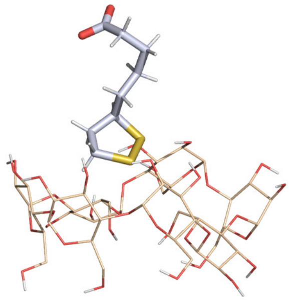

representative β-CD:ALA$^-$ structure

interaction free energy

ΔG_{INT} = –3.7 kcal mol^{-1}

γ-CD:ALA$^-$ COM graph

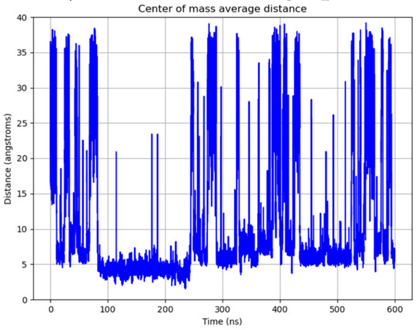

average COM value = 10.5 Å

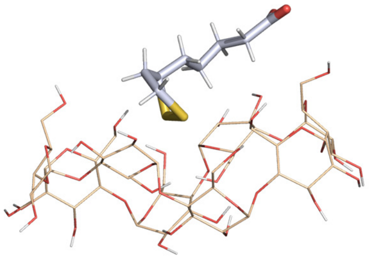

representative γ-CD:ALA$^-$ structure

interaction free energy

ΔG_{INT} = –8.5 kcal mol^{-1}

Figure S3. Representative structures of complexes between ALA$^-$ form and natural CDs in intestinal environment following 600 ns of molecular dynamics simulations **initiated from the well-separated components**. Data also include the evolution of the center-of-mass (COM) distances between components and their average values, together with the interaction free energies as obtained with the MM-PBSA analysis.

α-CD:ALA$^-$ COM graph

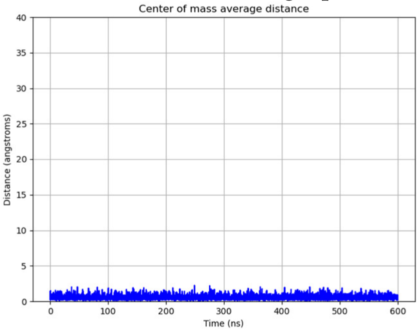

average COM value = 0.5 Å

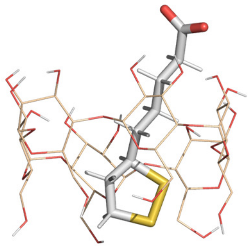

representative *α*-CD:ALA$^-$ structure

interaction free energy

ΔG_{INT} = –19.4 kcal mol^{-1}

β-CD:ALA$^-$ COM graph

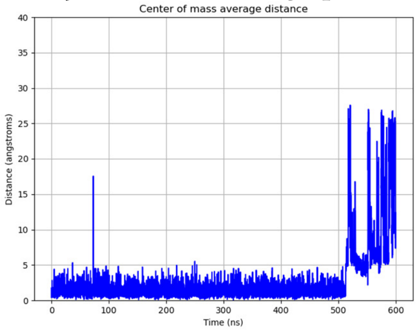

average COM value = 2.6 Å

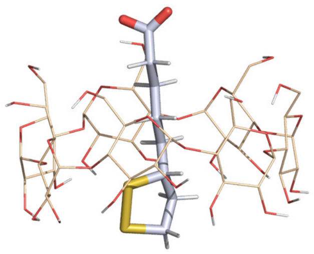

representative β-CD:ALA$^-$ structure

interaction free energy

ΔG_{INT} = –17.0 kcal mol^{-1}

γ-CD:ALA$^-$ COM graph

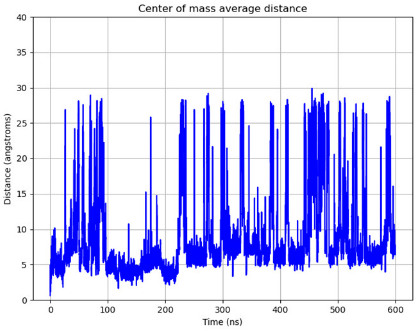

average COM value = 8.6 Å

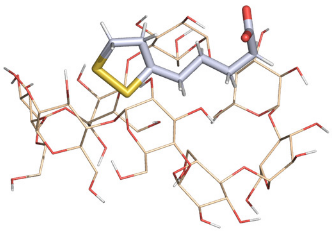

representative γ-CD:ALA$^-$ structure

interaction free energy

ΔG_{INT} = –8.4 kcal mol^{-1}

Figure S4. Representative structures of complexes between ALA$^-$ form and natural CDs in intestinal environment following 600 ns of molecular dynamics simulations **initiated from manually formed inclusion complexes**. Data also include the evolution of the center-of-mass (COM) distances between components and their average values, together with the interaction free energies as obtained with the MM-PBSA analysis.

HP-α-CD4:ALA⁻ COM graph	HP-β-CD4:ALA⁻ COM graph	HP-γ-CD4:ALA⁻ COM graph
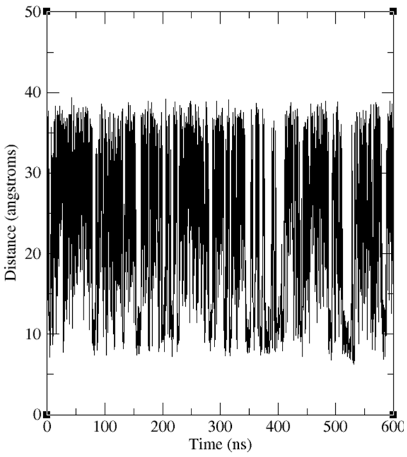	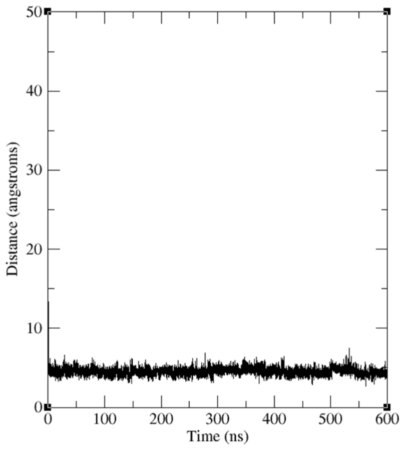	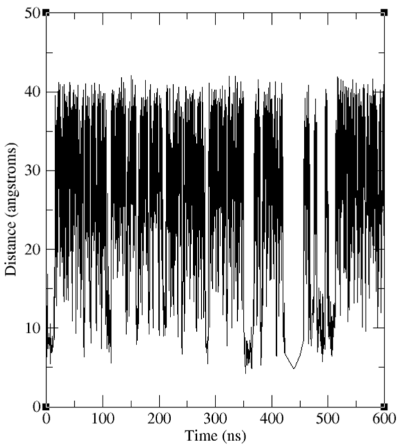
average COM value = 22.9 Å	average COM value = 4.5 Å	average COM value = 24.0 Å
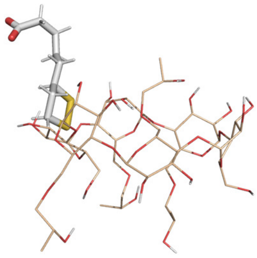	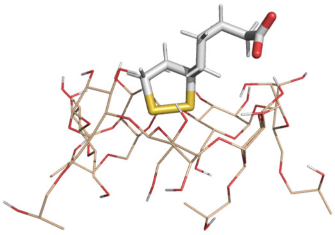	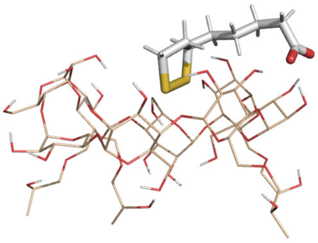
**representative HP-α-CD4:ALA⁻ structure**	**representative HP-β-CD4:ALA⁻ structure**	**representative HP-γ-CD4:ALA⁻ structure**
interaction free energy **ΔG_{INT} = –1.2 kcal mol⁻¹**	interaction free energy **ΔG_{INT} = –20.4 kcal mol⁻¹**	interaction free energy **ΔG_{INT} = –2.4 kcal mol⁻¹**

Figure S5. Representative structures of complexes between ALA⁻ form and tetrasubstituted HP-CDs in intestinal environment following 600 ns of molecular dynamics simulations **initiated from the well-separated components**. Data also include the evolution of the center-of-mass (COM) distances between components and their average values, together with the interaction free energies as obtained with the MM-PBSA analysis.

HP-*α*-CD_4:ALA^- COM graph

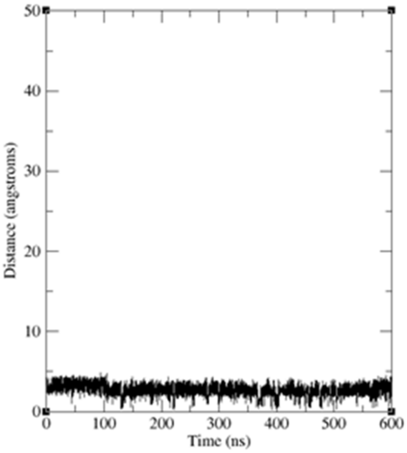

average COM value = 2.7 Å

HP-β-CD_4:ALA^- COM graph

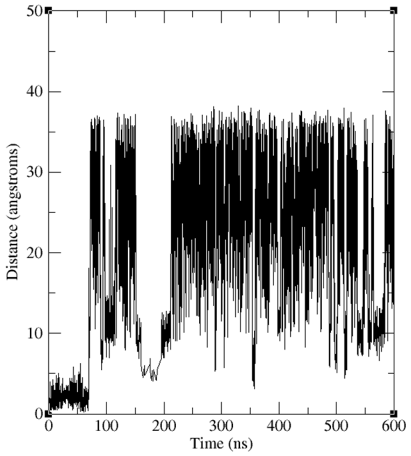

average COM value = 18.9 Å

HP-γ-CD_4:ALA^- COM graph

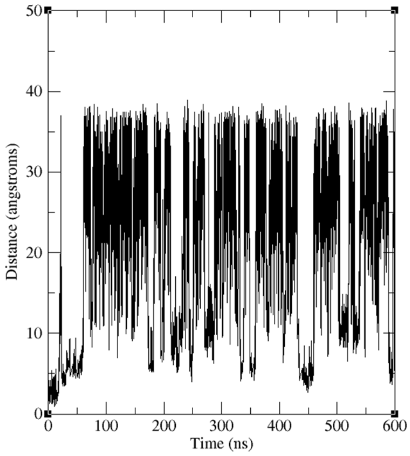

average COM value = 19.6 Å

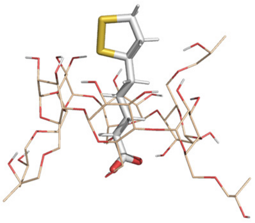

representative HP-*α*-CD_4:ALA^- structure

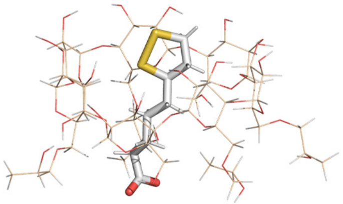

representative HP-β-CD_4:ALA^- structure

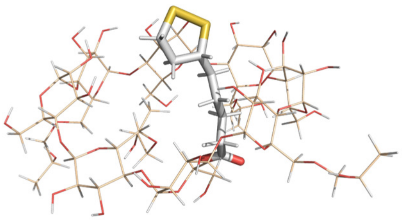

representative HP-γ-CD_4:ALA^- structure

interaction free energy **ΔG_{INT} = –21.6 kcal mol⁻¹**

interaction free energy **ΔG_{INT} = –4.6 kcal mol⁻¹**

interaction free energy **ΔG_{INT} = –3.9 kcal mol⁻¹**

Figure S6. Representative structures of complexes between ALA^- form and tetrasubstituted HP-CDs in intestinal environment following 600 ns of molecular dynamics simulations **initiated from manually formed inclusion complexes**. Data also include the evolution of the center-of-mass (COM) distances between components and their average values, together with the interaction free energies as obtained with the MM-PBSA analysis.

Table S1. UV stability of HPβALA prepared by different drying methods (spray-drying and lyophilization).

sample	ALA (mg/L)
**ALA (UV protected)**	100,5 ± 0,330
**ALA (UV treated)**	27,0 ± 11,3
**HPβALAsd-inclusion complex (UV protected)**	120,4 ± 0,833
**HPβALAsd-inclusion complex (UV treated)**	42,2 ± 27,9
**HPβALA-lyo-inclusion complex (UV protected)**	88,5 ± 49,0
**HPβALA-lyo-inclusion complex (UV treated)**	-5,3 ± 0,113
